# The Experience of Parkinson's Disease: A Systematic Review and Meta-Ethnography

**DOI:** 10.1155/2014/613592

**Published:** 2014-11-30

**Authors:** Andrew Soundy, Brendon Stubbs, Carolyn Roskell

**Affiliations:** ^1^School of Sport, Exercise and Rehabilitation Sciences, University of Birmingham, Birmingham B15 2TT, UK; ^2^School of Health and Social Care, University of Greenwich, London SE9 2UG, UK

## Abstract

There has been little attempt to summarise and synthesise qualitative studies concerning the experience and perception of living with Parkinson's disease. Bringing this information together would provide a background to understand the importance of an individual's social identity on their well-being and hope. Three primary aims were identified (a) understanding the importance of social identity and meaningful activities on individuals' well-being, (b) identifying factors and strategies that influence well-being and hope, and (c) establishing a model that relates to an individual's hope and well-being. Three stages were undertaken including a traditional electronic search, a critical appraisal of articles, and a synthesis of studies. Qualitative articles were included that considered the experience of living with Parkinson's disease. Thirty seven articles were located and included in the review. Five themes were identified and the themes were used to inform development of a new model of hope enablement. The current review furthered understanding of how physical symptoms and the experience of Parkinson's disease affect the individual's well-being and hope. Social identity was established as a key factor that influenced an individual's well-being. Being able to maintain, retain, or develop social identities was essential for the well-being and hope of individuals with Parkinson's disease. Understanding the factors which prevent or can facilitate this is essential.

## 1. Introduction

Individuals with Parkinson's disease have a reduced health-related quality of life and increased psychological and physical morbidity [1]. The symptoms of Parkinson's disease can be described as nonmotor (including psychological problems, cognitive impairment, or sleep disturbance) and motor (e.g., slowness of movement, rigidity in movement, experiencing tremors) symptoms [2]. Scientific understanding around the physical symptoms of Parkinson's disease is now well established and research is needed to focus more on the impact of the disease and the major challenges faced by individuals who have the diagnosis [3]. As part of this, psychosocial difficulties can be extremely challenging for individuals with Parkinson's disease, for instance, restriction in participation of activities, reduced social contact, or the inability to retain a job [4]. Recently, research has been able to link psychological morbidity to the physical symptoms and experience of living with Parkinson's disease. For instance, fluctuations in disease symptoms, severity of motor symptoms, and the duration of the disease have been associated with depression and anxiety [5]. This is supported by other evidence, for instance, the symptom of fatigue has been associated with a reduced quality of life as well as worse psychosocial behaviours [6]. Importantly, it has been identified that activities of daily living are a significant predictor of health-related quality of life for individuals with Parkinson's disease [7]. Further, recent research has identified that the physical aspects of the disease (e.g., a reduction in the ability to perform activities) combined with other determinants (e.g., stress, anxiety, and communication difficulties) decrease an individual's social activities, increasing isolation and leading to depression [4].

A negative effect on the meaningful activities that individuals undertake will also negatively influence their social identities.

Social identity is defined as the individual's knowledge that they belong to a certain social group, alongside the personal significance and emotional attachment of having that group membership [8]. Within rehabilitation settings, individual's goals are linked to, and formed by, their previous social identities [9]. However, an individual's ability to access their goals may be influenced by a number of factors which may challenge them and the success of any strategies which they may use to help overcome such challenges. For instance, how the illness is perceived to “biographically interrupt” their lives [10] may influence an individual's response to their illness and the resultant symptoms. Alternatively, other factors (e.g., the ability to accept the illness and subsequent changes in their life and social identities), as well as strategies used by individuals (e.g., using mental rehearsal to help accomplish tasks during rehabilitation) may influence an individual's ability to stay and remain hopeful [11].

It is possible that individuals with Parkinson's disease engage in activities which resonate with their social identity/ies and sense of belonging, strengthening a sense of who they are and providing a sense of purpose and satisfaction with life. Given this, it is important to acknowledge that an individual's social identities are likely to be strongly associated with their mental well-being (defined as satisfaction, optimism and purpose with life, a sense of mastery, control, belonging, and perceiving social support [12]) and generalised hope (defined as hope that exists on different levels, relating to the hope for relief in suffering and hopes which relate to an individual's social identities, meaningful and superficial activities, accomplishments, tasks, or interactions [13]). Thus, being able to (re)initiate social identities and meaningful activities is important as this has been found to aid mental well-being and generalised hope after diagnosis or injury [14].

A qualitative synthesis of studies would provide a way of understanding the importance of an individual's social identities and the subsequent factors and strategies that influence generalised hope and mental well-being. The previous value of qualitative findings has been illustrated by van der Bruggen and Widdershoven [15] who utilised novels to reveal the experience of patients with Parkinson's disease. However, their research did not consider a synthesis of experience; rather it provided a more descriptive account which lacked support from previous psychosocial theory/ies. A more recent study by Hartley et al. [4] was able to identify some barriers and facilitators related to psychosocial difficulties based on the analysis of 11 qualitative articles. However, only one paragraph was devoted to the results identifying a very brief description of barriers and facilitators to mental well-being. Thus, a synthesis which is broader in scope and focused on the experience of individuals with Parkinson's disease is needed. Further to this, given the recent research around the concept of generalised hope, social identity/ies, and mental well-being in other neurological research [9, 11, 13], there is reason to believe that qualitative data considering the experiences of individuals with Parkinson's disease can yield important insights and may further the current understanding around these concepts.

Within Parkinson's disease, there is extensive qualitative literature that considers the individual's experience and it is essential that this information is summarised in order to get an overview of literature. A synthesis of this literature would provide confidence in, and theoretical saturation of, the themes generated. It is possible that synthesizing this data would generate new understanding and further knowledge. Given the above, the purpose of the current study was to review the qualitative literature that considers the experience of living with Parkinson's disease and answer three primary research questions. (a) Are generalised hopes important for individuals with Parkinson's disease? (b) What factors and challenges exist to challenge or enable generalised hope following the disease onset? And (c) can a model that relates to an individual's generalised hope and mental well-being be established from aggregating the experience of individuals with Parkinson's disease?

## 2. Method

A metaethnography was chosen as it is able to synthesise information and further knowledge, often in the form of models that are generated [16]. As we wanted to generate a model, the primary author took a “subtle realist” approach to the review [17, 18]. This was important as, whilst we acknowledge the importance of individual findings from studies, it was believed that central psychosocial constructs (represented as underlying “truths” or reality [17]) can be related and represented within multiple study findings.

Traditionally, a metaethnography is undertaken in seven stages [19]; a detailed consideration of these stages can be considered in our previous work [16]. More recently metaethnographies have been reported within three phases [20], since this provides a simpler structure for the reader to follow. Within the current study we undertook a systematic review and metaethnographic synthesis in 3 phases: (1) a systematic search of the literature, (2) a critical appraisal of identified studies, and (3) a thematic synthesis of research to reveal overarching and emerging themes, as well as the development of a model of hope and well-being.

### 2.1. Phase 1: Systematic Search and Eligibility Criteria

A systematic search of major electronic databases (including AMED, CINAHL Plus, Medline, Embase, PsychINFO, and SPORTDiscus) was conducted by the primary author from inception until June 2014. The key search terms included perception OR optimism OR expectation OR hope OR identity OR self ∗ AND Parkinson's OR Parkinson's disease AND qualitative AND experience AND understand OR knowledge OR attitude AND interview OR focus group. In addition, we conducted hand-searching of the included articles' reference lists. The primary authors screened the titles of all identified articles. An article was included when it was considered that it satisfied all eligibility criteria considered within the domains of the SPIDER search tool [21].

*Sample*. Only individuals with a diagnosis of Parkinson's disease were included (both self-reported and medical diagnosis were acceptable). Studies with mixed samples were included if separate analysis on individuals with Parkinson's disease was given. 

*Phenomenon of Interest*. Articles were included if they were able to identify the experience of living with Parkinson's disease or able to identify factors which may influence the well-being or hope of individuals with Parkinson's disease. Articles were excluded if (a) they considered experiences related to a specific intervention or service which did not reflect usual care (e.g., if qualitative interviews were undertaken as a subsample from a randomised control trial or reporting the experiences of using deep brain stimulation); (b) they focused on caregivers or other stakeholders and did not consider the perspective of the patient with Parkinson's disease. 

*Design*. Any type of qualitative design was considered including phenomenology, grounded theory, or ethnography. Articles were excluded if they were case studies or reflective pieces, quantitative research, reviews, books, theses, or conference proceedings. 

*Evaluation*. Articles were required to include interviews or focus groups and document experiences, views, or attitudes from users, health care professionals, or carers (regarding the user, as opposed to personal experience) and were published in English.

*Result Type*. To be included, articles had to contain qualitative results which may include mixed methods studies.

### 2.2. Phase 2: Critical Appraisal of the Included Studies

The supervising author used the consolidated criteria for reporting qualitative studies (COREQ) [22] to assess the quality of the included studies. To ensure consistency in the approach to appraisal, the primary author randomly selected and appraised (blind) three articles (96 items). The kappa statistic between authors was *k* = 0.55 (*P* < 0.001). Following this, both authors considered where differences lay and adapted responses to questions, creating an agreed criterion for assessment. Differences in author answers mainly occurred through being more or less stringent when applying the requirements of an item from the COREQ to an included article. For instance, item 8 considers if authors identified the participant knowledge of the interviewer. This answer could be given a positive score or 1 point if authors identify the term “informed consent”; alternatively a more stringent criterion could require authors to explain and describe the relationship between participant and interviewer. The final approved COREQ scoring system used for appraisal is available from the primary author. The assessment of quality was provided by identifying a summary score calculated from each of the three COREQ domains. Domain 1 is entitled research team and reflexivity and contains 8 questions including the researchers personal characteristics (*n* = 5) and the relationship developed with participants and disclosure of research focus (*n* = 3). Domain 2 is entitled study design and contains 15 questions including the theoretical framework, sampling approach, sample size, and number of nonparticipation (*n* = 4), the setting of interest, and context (*n* = 4), and finally the data collection including the process of interviews, the use of different methods to capture data, and items relating to trustworthiness (*n* = 7). Domain 3 is entitled analysis and findings and contains 9 questions; this includes the data analysis and description of analysis processes (*n* = 5) and reporting of analysis (*n* = 4). The domains are combined to produce a total score. There are 32 items of which each is scored on the question either being reported correctly (scoring a point) or not (scoring no point), with a maximum possible score of 32. This tool has been used effectively in recent research [9] and aids confidence in the review's conclusion as well as potentially justifying the need for a review. For example, frequently, qualitative studies considering stroke patients experiences lacked the use of theoretical saturation and often did not develop minor themes [13]. Studies scoring a total of less than 16/32 (*n* = 3) were checked by the author BS for inclusion and studies scoring less than 12/32 (*n* = 0) were automatically excluded from the synthesis.

### 2.3. Phase 3: The Synthesis

The primary author undertook a thematic synthesis of the included studies [17]. The synthesis was “data driven” [23] initially in order to generate initial categories. After the analysis of half the included articles, the first draft of themes was considered, checked, and changed for repetition, thematic organisation and structure, and renaming themes subthemes and codes. Themes were considered for reciprocal (similar) and reputational (findings which refute) findings as well as line of argument synthesis (translating and bringing findings together) [24]. After this, the remaining articles were added in before reconsidering, checking, and changing themes. An audit trail is available from the first author. The model was developed using “idea webbing” (spider diagram to inspect and generate association between concepts) and “concept mapping” (the use of evidence from multiple studies to clearly identify the concepts with the generate model) [17, 25]. For the purposes of this article, only codes that contained at least 4 supporting articles are reported in this text. The reason for this was to focus on the most common and consistently reported themes, to increase the chance of the code being saturated theoretically and to reduce the size of the analysis, allowing for word limits within publication. Supplementary tables, however, report all codes.

## 3. Results

### 3.1. The Systematic Search

**Figure 1:**
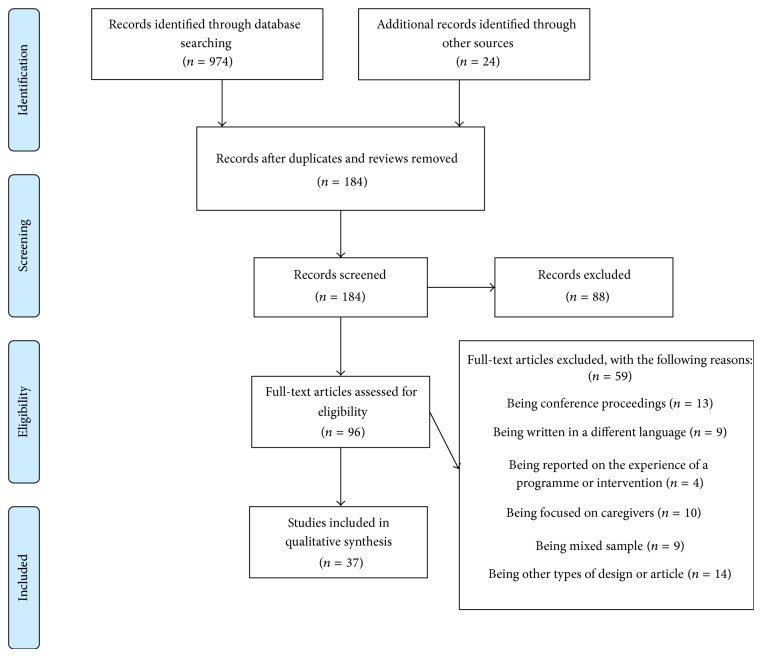
A PRISMA diagram for the review.

In total, 97 full-text articles were retrieved, of which 59 were excluded with reasons. A PRIMSA [26] flow diagram (see Figure 1) provides full details of the selection process. For full details of the excluded studies please see Supplementary File A in the Supplementary Material available online at http://dx.doi.org/10.1155/2014/613592 provides details of the excluded articles. A total of 37 articles [27–63] were identified from 36 data collections within 10 countries (the majority taking place in the UK = 13, USA = 11, and Sweden = 4). Across the studies, 582 individuals (male = 246, female = 192, unknown = 124) with Parkinson's disease were represented. The mean age across studies ranged from 51.6 years to 74.1 years, although 14 studies had a mean age of 70 ± 5 years.  Table 1 provides further details of participant characteristics.

### 3.2. Critical Appraisal of Studies

The COREQ [22] revealed that no studies were fatally flawed [64], meaning that the data presented in the results were not questionable and detailed well enough to be authentic as well as the methods and analysis documented to an extent that appropriate qualitative techniques had been undertaken.  Table 2 provides full details of the appraisal.

#### 3.2.1. Appraisal between Studies

The only three studies scored 16 or less (16/32, 50%): Banks and Lawrence [29], scoring 14; van der Eijk et al. [60], scoring 15; and Habermann [44] scoring 16.

#### 3.2.2. Appraisal within COREQ Domains

Domain 1, which considered the details of the research and reflexivity, scored well. However there were consistent problems identified within the other two domains. Within domain 2, which considered study design, most studies did not consider theoretical saturation (*n* = 31/37), did not take field notes (*n* = 31/37), or return transcripts to individuals (*n* = 27/37). Further, many (*N* = 16/37) did not position themselves in terms of their theoretical orientation. Within domain 3, studies consistently did not perform member checking (*n* = 28/37) and did not report minor themes or identify and discuss diverse cases (both within one item, *n* = 25/37).

### 3.3. The Synthesis

The synthesis generated 5 themes: (1) the effects of Parkinson's disease on an individual's social identity, (2) the psychosocial challenges and adjustment to Parkinson's disease, (3) factors that influence the severity of the interruption on individuals' life from Parkinson's disease, (4) cognitive, physical, and spiritual strategies and factors that influenced an individual's well-being, and (5) social support and interactions that influenced well-being. The themes were used to inform development of a model of hope enablement (a model which illustrates the importance of positive social identity/ies in promoting positive mental well-being and identifies the influencing factors which affect the (re)enablement of these identities and therefore mental well-being). Due to the length of the tables, they are presented in Supplementary File B (online) and include verbatim quotes as examples for the reader.

#### 3.3.1. Theme 1: The Effects of Parkinson's Disease on an Individual's Social Identity

Two subthemes were identified within this theme. The first was entitled the effects of illness on an individual's social identity/ies. Individuals described varied effects on their social identities and roles. Four responses from participants were given which included the following. (1) An unchanged core sense of who they are [31, 38, 40, 43, 44, 57]: for instance, some individuals were able to identify that certain identities such as those within the family or within a job (if it continued) remained unchanged although some individuals could also recognise that they were the same person, except for Parkinson's symptoms. (2) Individuals also highlighted the importance of retaining their old identities [35, 36, 38, 41–44, 47, 58], through retaining “normalcy” and distancing themselves from other identities like that of a Parkinson's “patient.” Individuals could emphasise the need to continue social identities and roles whilst fighting against the symptoms of Parkinson's disease. (3) Some individuals emphasised the effects of Parkinson's disease on their social identities [34, 35, 38, 43, 44, 46, 51, 57]. Individuals identified stereotypical characteristics linked to Parkinson's disease which they could be identified with, and in social situations this could represent their sole identity. However, they could also differentiate how they felt in their mind about themselves and what their body represented to others (4). A number of studies identified the loss and alteration of the individual's social identity [29–31, 34, 37, 38, 40–43, 45, 46, 49, 50, 53, 55, 57, 58, 61, 62]. In worst case, this meant knowing or remembering who they used to be. The losses were extensive and related to different roles like being a mother, or having an occupation, or more generally including losses in femininity or masculinity.

The second subtheme considered the effects of Parkinson's disease on individuals' activities and function. The effect on meaningful activities was essentially linked to the loss of social identities, as seen where individuals described activities they used to do as part of their identity, for example, being able to go shopping, to cook, and to provide for the family as a mother [27, 34, 38, 40–43, 45, 47, 49–51, 56–58]. Losses in function related to meaningful activities were also seen but also included taken-for-granted activities associated with independence like negotiating the stairs, putting clothes on, or being able to wear heeled shoes [34–36, 38, 40, 42, 43, 45, 47–49, 53, 54, 57, 61, 62].

#### 3.3.2. Theme 2: The Psychosocial Challenges and Adjustment to Parkinson's Disease

The two subthemes identified within this theme included the psychosocial challenges and psychological adjustment to the disease. The subtheme entitled the psychosocial challenges included five codes: decreased social confidence, self-esteem and competence, and being self-conscious. These decreases were attributed to a loss of social roles and activities combined with the symptoms of Parkinson's disease and related factors (such as the inability to communicate or the fear of falling). Individuals reported social anxiety as a result which impaired an individual's quality of life [29, 30, 34, 38, 40–42, 44, 46, 48–52, 55, 56, 59, 61, 62]. Part of an individual's social anxiety was linked to the perception of stigma in social situations, the stigma directly related to the disease symptoms and metaperceptions of what other people thought or said about them [29–32, 38, 39, 41, 42, 46, 48, 49, 51–53, 55, 57, 61]. Individuals described paranoid thoughts regarding their metaperceptions, a sense of embarrassment generated from their symptoms and being seen in public and social withdrawal as a result. The last three included increased levels of anxiety and stress as well as panic attacks [27–29, 32, 34, 39, 40, 55, 61, 62], a great deal of frustration towards the inability to complete activities of daily living independently [34, 41, 45, 47, 51, 54, 55, 57, 59, 61–63], a loss of control and uncertainty about life, and how Parkinson's disease would dictate an individual's functioning and ability to interact. Most individuals relied on medication to be able to continue. Further to this, individuals expressed a lack of control over motor activities as well as what the future will be and how the disease will progress [31, 34, 36, 37, 42, 43, 45, 47, 49, 54, 55, 57, 58].

The second subtheme considered how individuals adjusted to having Parkinson's disease. Three codes were identified within this subtheme. The first and second codes considered difficulty in coming to terms with the disease, primarily not accepting what had happened or being in denial [35, 41–43, 46, 47, 50, 51, 57, 62] but also because of the feelings of negativity, pessimism, depression, and chronic sorrow that could be exhibited [30, 37, 38, 43, 45, 47, 53, 54, 56, 58, 63]. Finally, individuals mentioned acknowledgement and acceptance [31, 35, 37, 38, 40–43, 45–47, 50, 51, 57, 58, 62]; individuals initially described acknowledging that something was wrong before diagnosis, then acknowledging and accepting the diagnosis, and then further being able to accept the different meaning it had for each person's life, including social and vocational identities.

#### 3.3.3. Theme 3: Factors That Influence the Severity of the Interruption on Individuals' Life from Parkinson's Disease

Three subthemes were identified including illness-related factors which affected individuals, psychosocial and situational factors which challenged well-being and environmental factors.

Illness-related factors that influenced individuals included the severity of the symptoms [29, 31, 34, 38, 47–51, 56]; the more severe the symptoms are, the greater the challenge was to be able to cope with and maintain a positive outlook. Second, individuals reported fluctuation in symptoms and notably “on” and “off” periods related to medication which meant there were times when movement or engaging in life would be impossible [29, 30, 34, 35, 38, 39, 41, 47, 48, 55, 62].

There were three clear codes that related to the psychosocial and situational factors which could challenge individuals; these included, first, the challenge of isolation in individuals' lives [29, 36, 38, 49, 51, 53–56, 61, 62], which was primarily influenced by a loss of activities and function and compounded by social anxiety and reduced self-esteem and confidence. Second, individuals were very aware of the burden they could become on others as Parkinson's disease forced a change of roles and increased their dependency on others, which could be intrusive at times, for instance, changing an individual's sanitary towel [29, 30, 34, 36, 38, 40–43, 46, 49, 50, 52, 54, 55, 57, 59, 62]. Third, individuals were very aware of their prospects in the future [34, 35, 37–39, 41–43, 45, 46, 48–50, 54, 58, 62], often from seeing others and often being a future that they had not planned for or considered which revolved around losses. In addition to this, individuals could worry about falling. Finally, individuals identified the difficulty in communication with others [27, 29, 38, 46, 49, 55, 57, 61]; for instance, individuals experienced changes in their voice, having a monopitch, but also were unable to speak coherently and could mumble, compounded by not being able to say what they were thinking. This in turn fostered a sense of insecurity and decreased their social confidence and willingness to engage.

#### 3.3.4. Theme 4: Cognitive, Physical, and Spiritual Strategies and Factors That Influence an Individual's Well-Being

This theme was the largest and most detailed theme containing 3 subthemes. These included (1) psychological and cognitive approaches to aid functioning and well-being, (2) physical and functional strategies that influenced well-being, (3) spiritual strategies that influenced well-being.

The first subtheme included 18 codes; they included the following. (a) In order to enhance the ability to move, individuals identified the importance of concentrating on movement, using self-talk and the mental rehearsal of movement [36, 43, 48, 52, 61]. (b) Individuals identified the benefits of bringing the future closer by living one day at a time or engaging in the present and not thinking too far ahead [41–43, 46, 51]. (c) Individuals identified the importance of making the best of their current situation which meant doing activities and continuing the best they could within the limitations imposed by the illness [30, 31, 37, 42, 43, 45, 46, 51, 57, 58, 61, 62]. (d) Individuals identified the importance of being positive and having a positive outlook which could influence well-being and act against a depressive outlook [30, 35, 36, 38, 41, 42, 46, 51, 54, 57, 61]. (e) Individuals identified having hope or optimism for the future as an important way to live, because without it, life could be very hard [30, 38, 42, 46, 55]. (f) Individual's hope varied, for instance, some may hope for a “good” outcome from rehabilitation or for achieving goals, whilst others had a more concrete hope for a miracle cure [42, 46, 47, 51, 55]. (g) Some individuals reported the importance of appreciating what they did have, including their current health and the ability to live the way they are or have the support that was made available to them [29, 30, 35, 41, 46, 47, 55]. (h) Individuals could make comparisons to others in worse situations and be thankful for their current situation [31, 34, 37, 40, 41, 46, 51, 57, 61, 62]. (i) The importance of humour was identified as an important outlet for living with and experiencing Parkinson's disease [31, 38, 46, 52]. (j) Individuals identified that it was important to retain their dignity, which meant maintaining a social identity and managing how others saw them; this included being seen as they used to be (past identity) or retaining dignity in social settings [38, 41, 42, 57]. (k) A number of individuals identified the importance of being resilient against Parkinson's disease. Individuals identified that they would not give up and had strong and distinguishing characteristics which enabled this, like exceptional determination [31, 35, 38, 40, 41, 50–52, 55, 57, 58, 63]. (l) Some individuals chose not to reveal, or to actively hide, the diagnosis from others by undertaking behaviours which avoided individuals observing their symptoms [31, 36, 41, 44, 46, 47, 54, 56, 57]. (m) Individuals could actively stop thinking about the disease and their future and focus on living normally [31, 35, 37, 44, 50]. (n) Learning more about their disease was considered very important and sources of information included health care professionals, Parkinson's disease support groups, and family members seeking information on their behalf [32, 36, 38, 41, 43, 44, 46]. (o) Generally a great number of individuals wanted to maintain autonomy and their independence; this was important if they were to maintain their identities and meaningful activities [38, 41, 42, 44, 46, 47, 50, 51, 54, 59, 63]. (p) Identifying a manageable routine and planning what could be achieved was essential for individuals as the disease often required them to take rest periods in the “off” times or fatigue prevented them doing too much [31, 36, 38, 40, 42, 43, 47, 51, 55, 57, 61–63]. (q) Some individuals identified the value of looking after the needs of others and, as a result of having Parkinson's disease, became more giving, for instance, by volunteering at Parkinson's society [36, 38, 41, 45, 47, 50, 57]. (r) Finally, individuals identified the importance of adapting activities in order to continue engagement and interactions which could include leisure pursuits, hobbies, meeting with groups, and family [29, 32, 35, 38, 40, 41, 43, 44, 51, 57, 62].

The second subtheme identified four codes; these included first the importance of individuals living normally despite their Parkinson's disease, as this enabled an individual to maintain confidence, social contact, and a sense of purpose [35, 36, 40, 42–44, 52]. Second individuals identified the importance and need to manage their movement and actions, through taking their time over the movement and understanding the need for rest in order to perform and function [35, 36, 38, 40, 44, 45, 47, 52, 54, 55, 57, 62]. Third, individuals identified a range of physical and functional aids that could improve living, for instance, adaptions to the home like a walk-in bath or support rails, using an electronic tooth brush or “dosette” boxes [28, 29, 35, 37–39, 62, 63]. Finally, some individuals recommended the use of exercise and other alternative therapies [38–40, 42, 46, 47, 54, 61, 62].

The final subtheme considered spiritual factors which influenced an individual's well-being, these demonstrating the importance of faith in helping these individuals accept what was happening, cope with what was happening, and be hopeful in the knowledge that God had a plan and purpose for their life [30, 32, 45, 46, 50, 56, 57]. Second, individuals utilised prayer for different reasons including managing the disease, making decisions (e.g., financial ones, knowing what activities to engage in), or using prayer for the purposes of healing [31, 35, 38, 39, 53].

#### 3.3.5. Theme 5: Social Support and Interactions That Influenced Well-Being

Two subthemes were identified within this theme. These included the use and benefits of social support and the experiences of interactions with health service personnel.

Seven codes made up the first theme; firstly individuals acknowledged the overall importance of close social support from family [28–30, 32, 34, 36, 38, 41, 47, 51, 61, 63] and friends or others [28, 30, 32, 36, 38, 40, 41, 46–48, 50, 52, 63]. Second, individuals identified the importance of emotional support in that they could feel valued, accepted, and not judged or discriminated against because of their Parkinson's disease. Part of this social support required others to be informed about Parkinson's disease so that misunderstanding about behaviours and stigma were minimised and included being sensitive to the needs of the individual by being patient and understanding [38, 43, 47, 48, 50, 63].

Third, individuals identified the importance of belonging and having unity with others who had been diagnosed with Parkinson's disease. One reason for this is because they were able to relate to and understand the experiences of one another [32, 35, 36, 38, 39, 41, 42, 46, 51, 54, 55, 57, 60, 61]. Fourth, acts of tangible support were important including support from local government, friends, or family and revolved around tangible assistance like travel [28–30, 32, 38, 41, 43, 46, 48, 49, 53, 54, 60]. Fifth, individuals identified the importance of informational support in gaining access to information relating to Parkinson's disease, medication usage, and information which could aid daily living [30, 32, 38, 39, 43, 46, 48]. Sixth, individuals would value esteem support and encouragement from others as a source of motivation which could enhance the ability to cope [30, 34, 46, 48, 49]. Finally, individuals identified reasons why a lack of support could be apparent; these included (a) close others not being in a position to provide support for physical or emotional reasons, (b) feeling alienated and misunderstood by other people, (c) particular conflict identified which caused distress for individuals and families; this included not talking about their situation, arguing about it, or worst case getting separated as a result of it [30, 32, 34, 38, 40, 41, 43, 46, 47, 50, 52, 54, 56, 58, 61]. The second subtheme included one code which related to the positive experiences and importance of good health care professionals in helping the individual cope, being a source of confidence and demonstrating through communication that they valued the individuals [27, 32, 38, 39, 41, 46, 47, 56, 60, 63].

#### 3.3.6. The Hope Enablement Model (HEM)

**Figure 2:**
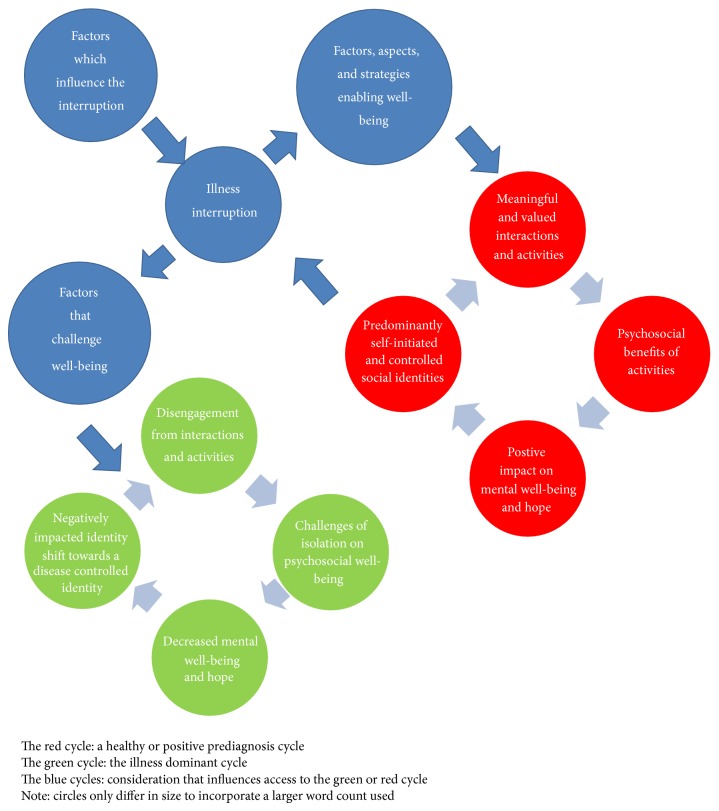
The model of hope enablement.

*(1) Model Summary and Outline*.  Figure 2 illustrates the hope enablement model. The model has been based on the central importance of social identity and meaningful activities identified in previous literature within different neurological populations [9, 13, 14, 65, 66]. It is based primarily on how generalised hope is understood from previous literature [13], which constructs hope by bringing together existing scholars [16, 67–71] considerations and understanding of what hope is and how it is constructed.

A healthy or positive prediagnosis cycle exists and is considered a central cycle to the model (illustrated in red). This cycle suggests that social identities are (to some extent) stabled and self-initiated and controlled, which leads to meaningful engagement in activities and interactions and in turn provides psychosocial benefits for individuals as well as hope and well-being. A general outline of how individuals may progress through the model suggests that, following the onset, change, or challenge of Parkinson's disease and related symptoms (labelled illness interruption), individuals either positively respond and are able to reengage in the positive cycle or negatively respond and face the challenges of a negative cycle.

The illness dominant cycle (illustrated in green) reflects a disengagement from meaningful interactions and activities and exacerbated negative effects from the illness identity (dominance of preexisting stereotypical illness-related traits typical of the disease) which creates isolation for the individuals and in turn this negatively influences the stability of their social identities, as well as their well-being and hope. This makes the individual more vulnerable to further decreases in well-being and the possibility of assuming a more disease influenced identity which is defined by stereotypes of the illness and symptoms presentation.

*(2) Factors Which Influence the Interruption*. Factors which influence the interruption can be identified from the current review within Section 3.3.3 and included how the illness has influenced their identity, if accessing a previous identity is possible, the extent of the symptoms, and the change experienced and the “on” and off periods of the illness. It is likely that other factors are also present such as the time since the change, the deterioration of improvement. These factors must also be considered as challenging an individual's well-being (Section 3.3.6(3)). 

*(3) Factors, Aspects, and Strategies That Enable Well-Being and the Ability to Engage in a Positive Cycle*. Individuals are able to challenge the interruption and using strategies to enable well-being leading back into a positive cycle and the benefits that are generated from that. Eighteen strategies were identified in Section 3.3.4 and existed across cognitive, physical, and spiritual domains. Factors which influence the individual's ability to engage in a positive cycle from the current research included: personality attributes, motivation, and willingness to change. This could be examined by participant's identification of their determination to challenge what has happened and emphasize that they were a “fighter” or were extremely resilient to what had happened. Further to this it has been identified that resilience is associated with less depression and greater optimism [72]. Another aspect that has been previously been associated with less emotional distress is seeking to understand more about the disease [73].

More broadly it is likely that Snyder et al.'s [74] hope theory fits within this category suggesting that agency (internal beliefs and motivation) and pathways (how individuals will achieve their goal) as well as goals play an important role for individuals in their ability to positively respond to their illness. Further to this social support was identified for its influence of well-being and likely has a role in enabling hope and a positive cycle reengagement for individuals with Parkinson's disease. This supports previous literature that suggests there is a beneficial influence of social support on anxiety and depression [75]. 

*(4) Factors and Aspects That Challenge Well-Being*. Individuals may negatively respond to the factors that are presented, which in turn leads to a negative cycle (green cycle). The factors which challenge well-being from the current review included decreases in social competence, confidence, and self-esteem, as well as being self-conscious in social situations and being vulnerable to social anxiety and the effects of stigma and the metaperceptions of others. Further to these factors, how individuals have adjusted to Parkinson's disease is important; for instance, individuals can be vulnerable to increased anxiety and panic attacks as well as perceiving a loss of control and a restricted ability to interact and engage in life. Further some could not come to terms with the illness or accept what it meant in their life or what losses it caused. Health care professionals also must be aware that poor interactions can challenge an individual's well-being [76].

## 4. Discussion

There was a great need for a synthesis of qualitative studies that have considered the experiences of patients who live with Parkinson's disease. This was needed in order to enhance the quality of care offered by healthcare professionals. The critical appraisal of studies highlighted methodological limitation of existing literature, particularly the lack of reference to theoretical saturation as well as a lack of detail around minor themes. The current review provides important theoretically saturated data from the analysis and confidence in the results obtained, especially within the minor themes (codes). The synthesis of evidence was able to identify all the aims of the review, identifying the importance of social identity/ies and meaningful activities on hope and well-being, revealing the factors and strategies that influence an individual's well-being and hope, and, finally, developing a simple hope enablement model which likely has universal application across other chronic neurological conditions as well as other chronic illnesses. For instance, all components of the model are supported in research considering stroke, spinal cord injury, and multiple sclerosis [9, 11, 13, 14, 16, 77] as a majority of components are identified in other chronic illness like schizophrenia [78], juvenile idiopathic arthritis [79], and chronic heart failure [80].

### 4.1. Understanding the Central Importance of an Individual's Social Identity/ies

The current results indicate that health care professionals, working with patients who have Parkinson's disease, should have better understanding of their patient's social identities and meaningful activities. It is important to recognise that the illness can intrude physically which in turn influences an individual's social identity or sense of self [81]. It is clear from the current findings that one's illness identity, generated from stereotypical deficits in functioning, fosters vulnerability towards social engagement. It is clear that being able to continue to be independent and engage in activities has great value for individuals and considering how an individual's social identity/ies can be maintained or adapted is an important part of considering what activities are important for the individual. For instance, an athletic identity can be maintained following spinal cord injury [14], a positive identity can be maintained following a stroke by using group membership [66], or alternatively a positive identity can be reclaimed by using artistic expression [82]. Given the above, clinicians should recognise the potential value of restoring or initiating social identities, often through activity groups, of patients with Parkinson's disease.

The model with a focus on identity was devised for simple, patient-centred application in clinical practice. Clinician's should consider how Parkinson's disease has impacted on their social identity/ies; acknowledging the severity and magnitude of this may vary tremendously. It may require clinicians to look into the narrative or story they hear from a patient [16]. This is important because the individuals stories can reflect their hope and adjustment, or what they can accept or need to defy given their illness and future prospects [77]. Honouring and understanding an individual's social identity/ies and how the illness has impacted them is one way to prevent a lack of empathy which may be created by a pressurised clinical environment [83, 84].

### 4.2. Recognising the Factors Which Influence and the Strategies Which Facilitate Well-Being and Hope

Individuals with Parkinson's disease may reduce their social contact as a direct result of their symptoms including reduced mobility and a lack of movements needed for everyday activities [4]. The current results were able to consider this further and identify the impact of Parkinson's disease on an individual's social confidence and social engagement. It has previously been recognised that better adjusted individuals with Parkinson's disease are able to undertake more activities [85]. The current results further this by identifying that health care professionals are required to look past the individual's ability to accept the diagnosis and consider wider adjustment, such as the ability to engage in society. It is important to recognise that poor physical functioning does not solely result in reduced engagement in activities; rather perceiving the effects of stigma and a reduction in social confidence may also play a role [78, 79]. It is important to acknowledge individuals' vulnerability identified within the current model (HEM) towards social disengagement and isolation; this would likely be associated with a decrease in activities of daily living and increased chances of depression. Given the importance of maintaining activities of daily living for quality of life [7] and the impact of depression on independence and activities of daily living [3], this, in terms of the HEM, is a central cycle to avoid.

### 4.3. Limitations

During the searching process it was clear that there were a great number of conference abstracts available that were not included; further there were a number of articles that were not included because they were not written in English (*n* = 9, 9/46; 20%). Given the high numbers of included articles it is difficult to determine what influence this had on the results, especially considering the theoretical saturation of some themes and codes, but it must be acknowledged that the representativeness of the patient group may be a factor which has influenced the current results and conclusions. The analysis focused on particular concepts and had a certain theoretical orientation (towards the theory of hope and well-being); as a result some of the uniqueness presented in articles may have been lost to the analysis. Further considerations of political and environmental factors were limited in the current data. It is important to acknowledge the potential for previous research to impact on the primary author analysis; for instance, whilst the analysis was data driven, recent research considering hope in individuals who have suffered a stroke may have influenced the choice of generic themes to use [11, 13]. The current average age of participants in the included studies may impact on the results and current understanding from this article. Finally, it is important to note the quality assessments of articles may be limited by the word limits given by journals; thus the COREQ score must be considered with this in mind.

### 4.4. Conclusion

The current review has identified the importance and influence of individual's social identities and meaningful activities on their well-being and hope. Further, that there are factors which challenge an individual's hope and well-being but also strategies that are employed to overcome such challenges. The current results provide a clear illustration of the current knowledge base and identify where some elements of understanding are well supported by literature and where other areas are not. This information may be useful to guide further research. It is important that clinicians consider the model proposed, utilising it to support and enhance patient-centred care and improving the well-being of patients with Parkinson's disease.

## Supplementary Material

Supplementary File A: Details of the 62 excluded articles.
Supplementary File B: The tables illustrating the full thematic analysis.

## Figures and Tables

**Table 1:** The study characteristics of included studies.

Study	Qualitative method	Participants	Sampling and setting	Data collection aims and focus
Anderson (2013) [27]	Semistructured interviews	13 (10♂) patients with Parkinson's disease Mean age: not given Median age: 66.5 years (range, 44 to 80 years) Time with illness: not obtained Hoehn and Yahr stage 1 = stage 2 8 = stage 3 4 = stage 4 1 = stage 5	Sampling: purposive (those having surgery) Setting: participants homes	Focus was on the preoperative experiences of care 14 surgeries, 10 = orthopaedic, 2 = cardiac, 1 = hernia, 1 = battery replacement

Andersson and Sidenvall (2001) [28]	Case studies	10 women with Parkinson's disease Mean age: 74.1 ± 4.1 years Median age: 75 years (range, 67–80 years) Mean time with illness: ≈8.7 ± 3.3 years Median time with illness: ≈10 years (range 4–14 years) Hoehn and Yahr stage: not given	Sampling: purposive or random (from geriatric and neurological clinic register) Interviews location: participants homes	Identify how women with Parkinson's disease managed to cook and shop and consider if their nutritional needs were met

Banks and Lawrence (2006) [29]	One-to-one interviews (likely semistructured)	24 (10♂) patients with Parkinson's disease Mean age: 51.6 years, no further details Median age: not given (range, 43–61 years) Mean time with illness: 4.2 years (in employment), 8.6 years (not in employment) Hoehn and Yahr stage: not given	Sampling: purposive (selected on the basis of gender, employment, and time since diagnosis) Interview location: not clear	Focus on assessing the impact on Parkinson's on employment

Benharoch and Wiseman (2004) [62]	Semistructured interviews, combined with a researcher diary and participant diary	8 (4♂) patients with Parkinson's disease Mean age: 3 over 65 years, 7 under 65 years, no further details given Median age: not given Mean time with illness: 9.2 ± 5.9 years Hoehn and Yahr stage: not given	Sampling: snowball sampling was used. Interview location: not clear	Focus on participation in self-care and the use of occupations and the meaning ascribed to them

Bingham and Habermann (2006) [30]	Individual and family semistructured interviews	56 participants with Parkinson's disease and primary caregivers Mean age of individuals with Parkinson's disease: 71 years (range, 41–87 years) Mean age of caregivers: 71 years (range, 44–88 years) Mean time with illness: not given (range, 6–9 years) Hoehn and Yahr stage Either stage 3 or stage 4 (no further details given)	Sampling: maximum variation sample Interview location: not clear	Consider if spirituality assists individuals with Parkinson's in the management of the day to day experiences of the illness

Bramley and Eatough (2014) [31]	Case study	1 female Age: 62 years Hoehn and Yahr stage 4	Sampling: case study Interview location: not identified	Three interviews consider life history, impact of diagnosis, issues of medication, and the future

Brigersson and Edberg (2004) [32]	Semistructured interviews	6 couples or 12 individuals (6 = ♂) (6 with Parkinson's disease, 3 = ♂) Mean age of individuals with Parkinson's: 71.2 ± 5.8 years (range, 61–75 years) Mean age of spouse: 74.3 ± 4.7 years (range, 66–82 years) Mean duration of illness: 11.8 ± 8.2 years Hoehn and Yahr stage not given	Sampling: purposive sample of couples (married or cohabiting) Interview location: respondents homes	Consider the support experience of individuals with Parkinson's disease and their partner

Buetow et al. (2012) [33]	Semistructured interviews	22 individuals (13 with Parkinson's disease, 9 = ♂) Mean age: not given Median age: not given (range not provided, age groupings, 6 = <65 years, 12 = 65–74 years, 4 = 75+ years) Means time with illness: 11.0 ± 5.6 years Hoehn and Yahr stage: not given	Sampling: maximum variation through national advertisement Interview location: telephone interviews	Explore the meaning and significance of patient safety events in individuals with Parkinson's disease. Site locations 15 = hospital or general practice, 7 = other places

Caap-Ahlgren et al. (2002) [34]	Semistructured interviews	8 women with Parkinson's disease Mean age: 70 years (range, 63–80) Mean time since diagnosis: not given (range, 5–15 years) Hoehn and Yahr stage Stage 1 = 1 Stage 2 = 2 Stage 3 = 3 Stage 4 = 1 Stage 5 = 1	Sampling: purposive sample of couples (range of age, duration of illness, civil status, and dwelling) Interview location: respondents homes	Explore experience of women living with Parkinson's disease.

Charlton and Barrow (2002) [35]	Semistructured interviews	8 (3 = ♂) participants with Parkinson's disease Mean age: 62 years (range, 62–86) Mean duration of illness: 3 years (range, = 3–8 years) Hoehn and Yahr stage: not given	Sampling: convenience sample Interview location: respondents homes, except one who was interviewed in hospital interview room	Consider the consequences of living with Parkinson's disease. Coping methods were considered that help psychological distress and if coping was related to group membership

Davis et al. (2003) [36]	Focus group interviews	9 individuals (6 = ♂) with Parkinson's disease Mean age: 70.6 ± 10.8 years (range, 51–85 years) Mean duration of illness: 9.8 ± 5.4 years (range, 3–20 years). Hoehn and Yahr stage: not given	Sampling: convenience sample (advertisement in local hospitals, outpatients rehabilitation, and community Parkinson's groups) Interview location: local church where the Parkinson's support group held their meetings	Focus on challenges to walking, the loss of balance and falls experience, general mobility, and exercise

Delaney et al. (2012) [37]	Semistructured interviews	10 individuals (7 = ♂) with Parkinson's disease Mean age: 56.8 ± 8.1 years (range, 49–70 years) Mean duration of illness: 8.4 ± 3.5 years (range, 4–15 years) Hoehn and Yahr stage: not given	Sampling: purposive with a focus on individuals with impulse control behaviours Interview locations: unclear	Consider the perspective of individuals with Parkinson's on impulse control behaviours

Den Oudsten et al. (2011) [38]	Focus group interviews	38 (24 = ♂) individuals with Parkinson's disease, 8 (6 = ♂) caregivers, and 8 (3 = ♂) health care professionals Patients with Parkinson's disease Pooled mean age: 67.4 ± 6.6 years Mean duration of illness: caregivers Pooled mean age: 55.0 ± 6.4 years Health care professionals Pooled mean age: 40.5 ± 7.5 years Hoehn and Yahr stage: not given	Sampling: purposive (adults 18–65 years) identified by neurologist Interview locations: multiple including educational facilities, outpatient clinics, and hospitals	Consider a qualitative understanding of what quality of life means for individuals with Parkinson's disease

Drey et al. (2012) [39]	Semistructured interviews	15 (9 = ♂) individuals with Parkinson's disease, 3 (? = ♂) carers Mean age: not given (range, 44–74 years) Mean duration with illness: not given (range, 1–17 years) Hoehn and Yahr stage: not given	Sampling: purposive (those responsible for managing their own medication with help of a carer) Interview location: hospital setting following routine clinic appointment	Consider the role and value of Parkinson's nurses for patients and some carers

Elliott and Velde (2005) [40]	Semistructured interviews	7 individuals (3 = ♂) with Parkinson's disease Mean age: not given (range, 55–79 years old) Mean duration of illness: not given (range, 3–18 years) Hoehn and Yahr stage: not given	Sampling: purposive and nominated sampling from Parkinson's support group Interview location: not clear	Consideration to the changes in lifestyle, the impact of Parkinson's on an individual's occupation and habits, and how occupation is integrated in to life

Fleming et al. (2004) [41]	Descriptive case studies	19 women with Parkinson's disease Mean age: 44 years (range, 34–56 years) Mean duration of illness: not given (range 18 months–27 years). Hoehn and Yahr stage: not given	Sampling: snowball (women with Parkinson's from four clinics who would then identify other potential participants) Interview location: study centre location (educational facility)	Experiences and adjustment made by women to Parkinson's in relationship to womanhood.

Habermann (1996) [43]	Semistructured interviews	16 (9 = ♂) individuals with Parkinson's disease Mean age: 48 years (range, 42–59 years) Mean duration of illness: not given (range not given, age groupings; 5 = < years, 4 = 5–10 years, 1 = 16 years, no other details given) Hoehn and Yahr stage “majority” stages 2-3.	Sampling: purposive (restricted aged range of individuals from “various” neurological practices) Interview location: not detailed	Consider the day-today experiences of middle aged individuals with Parkinson's

Harberman (1999) [44]	Semistructured interviews	16 (9 = ♂) individuals with Parkinson's disease Mean age: 48 years (range, 42–59 years) Mean duration of illness: not given (range, 1–16 years). Hoehn and Yahr stage “majority” stages 2-3	Sampling: purposive (restricted age range) Interview location: not detailed	Consider the challenge of Parkinson's to an individual's sense of self and ability to cope

Hermanns (2011) [45]	Interviews undertaken	14 (7 = ♂) individuals with Parkinson's disease Mean age: 68.4 years (range, 38–82 years) Mean duration of illness: not given (range, not given). Hoehn and Yahr stage Stage 1 = 2 Stage 2 = 2 Stage 3 = 3 Stage 4 = 6 Stage 5 = 1	Sampling: purposive (early onset and either over or under 60 years of age) Interview location: within a “support group community location” (presumably)	Consider the daily experience of Parkinson's disease

Hodgson et al. (2004) [46]	Semistructured interviews	20 individuals 10 (6 = ♂) individuals with Parkinson's disease Mean age: 61.8 years (range, 46–79 years) Caregiver mean age: 62.4 years (range, 52–79 years) Mean duration with illness: 7.5 years (range, 2–20 years) Hoehn and Yahr stage Stage 1 = 2 Stage 2 = 6 Stage 4 = 2	Sampling: purposive (couples with Parkinson's) Interview location: at couple home, except one couple who opted for a business location	Consider the impact of Parkinson's on the relationship of a couple

Hurt et al. (2012) [47]	Semistructured interviews	37 individuals with Parkinson's disease Mean age: not given Mean duration with illness: not given Hoehn and Yahr stage: not detailed	Sampling: purposive (nondepressed and depressed individuals age matched across groups) Interview location: not detailed	Experience and perception of Parkinson's disease and its relationship to depression within nondepressed, mild depressed, and moderate-severe depressed individuals.

Jones et al. (2008) [48]	Semistructured interviews	20 (12 = ♂) individuals with Parkinson's disease Mean age: 65 years (range, 50–80 years) Mean duration of illness: 10 years (range, 2.5–20 years). Hoehn and Yahr stage Stages 1–4 identified (no further details given)	Sampling: purposive (Hoehn and Yahr stage 1–4, no dementia, no severe dyskinesias, no long period making table testing difficult, no severe comorbidity, <80 years old, no medical or joint problems affecting mobility) Interview location: not detailed	The challenges faced by individuals with Parkinson's when attempting to walk each day

Liao et al. (2013) [49]	In-depth interviews	15 (9 = ♂) individuals with Parkinson's disease Mean age: 73 years (range, 65–80 years) Mean duration of illness: not given (range, 2–15 years). Hoehn and Yahr stage Stage 1 = 3 Stage 2 = 5 Stage 3 = 4 Stage 4 = 3	Sampling: purposive Interview location: not detailed	To consider the experiences of older Taiwanese individuals during different stages of Parkinson's disease

Lindgren (1996) [50]	Semistructured interview	6 (3 = ♂) individuals with Parkinson's disease Mean age: not given (range, 66–78 years) Mean duration of illness: not given (two reported a range of 2–4 years, four reported a range of 11–25 years). Hoehn and Yahr stage: not given	Sampling: purposive Interview location: not detailed	Identify long term grief or chronic sorrow experienced by individuals

Marr (1991) [51]	Interview (type not given)	6 (3 = ♂) individuals with Parkinson's disease Mean age: not given (range, 53–79 years) Mean duration of illness: not given (range, 2–15 years). Hoehn and Yahr stage Stages 1–2 “some” Stages 3-4 “a few”	Sampling: purposive (Parkinson's disease for at least a year, being able to understand English, independently ambulatory) Interview location: own homes	Considered the experience of living with Parkinson's disease

Miller et al. (2006) [52]	Semistructured interviews	37 (23 = ♂) individuals with Parkinson's disease Mean age: 70.9 ± 9.6 years (range, 50–88 years) Mean duration of illness: 9.5 ± 7 years (range, 3–38 years). Mean Hoehn and Yahr stage: 2.67 ± 0.9 (range, 1–5)	Sampling: purposive (sample had speech and swallowing problems) Interview location: own homes	Consideration of the onset and impact of speech changes and the strategies used to manage changes

Mshana et al. (2011) [53]	In-depth interviews and focus groups	34 individuals (32 = ♂) 28 (? = ♂) individuals with Parkinson's disease, 28 carers, 4 health workers, and 2 traditional healers (no further information given on these groups) Mean age: not given (range, 45–90 years) Mean duration of illness: not given Hoehn and Yahr stage: not given	Sampling: convenience/purposive (individuals within one district) Interview location: within community location (no further details given)	Perceptions and experiences of Parkinson's disease

Oehlberg et al. (2008) [54]	Semistructured interview	38 (32 = ♂) individuals with Parkinson's Mean age: 65.5 ± 12.1 (range, not given) Mean duration of illness: not given Hoehn and Yahr stage: not given	Sampling: convenience (individuals within one centre for Parkinson's disease that had depression) Interview location: not clear	Consideration for the preference of Parkinson's patient of the etiology of and preferred treatment strategies for depression

Olsson et al. (2013) [55]	Semistructured interview	11 women with Parkinson's disease Mean age: not give Median age: 59 years (range, 45–64 years) Mean duration of illness: not given Median duration of illness: 7 years (range, 1–13 years) Hoehn and Yahr stage: not given	Sampling: convenience (women identified from one hospital location) Interview location: 9 in own homes, 2 at university	Women's experiences of living with Parkinson's and fatigue

Pretzer-Aboff et al. (2009) [63]	Semistructured interview	3 (? = ♂) individuals with Parkinson's Mean age 78.7 ± 3.5 (range, 75–82 years) Mean duration of illness: 7.3 ± 7.0 years (range, 1–17 years)	Sampling: purposive (individuals identified from the local community) Interview location: a quite private place of an individual chosen	Learn about the barriers, facilitators, and help techniques used by individuals with Parkinson's to aid their functioning

Soleimani et al. (2014) [56]	Semistructured interview	11 (7 = ♂) with Parkinson's disease Mean age: 71 ± 10.5 years (range, 60–90 years) Mean duration of illness: 5.4 ± 4.2 years (range, 1–16 years) Hoehn and Yahr stage: 2.4 ± 1.1 years (range, 1–5)	Sampling: purposive (individuals identified from one hospital location) Interview location: in individuals own homes	Consider the effects of Parkinson's on social connections

Stanley-Hermanns and Engebretson (2010) [57]	Participant observation and in-depth interviews	14 (7 = ♂) with Parkinson's disease Mean age: 68.4 years (range, 38–82 years) Mean duration of illness: not identified Hoehn and Yahr stage: Stage 1 = 2 Stage 2 = 2 Stage 3 = 3 Stage 4 = 6 Stage 5 = 1	Sampling: purposive (individuals selected to represent a variation in severity of their disease and ability to articulate) Interview location: in individuals own homes	Consider how individuals with Parkinson's manage living with their illness on a day-to-day basis and how individuals construct their illness

Todd et al. (2010) [58]	Semistructured interview	8 (7 = ♂) with Parkinson's disease Mean age: 70.5 ± 5.6 years (range, 63–79 years) Mean duration of illness: 10.0 ± 4.5 years (range, 3–19 years) Hoehn and Yahr stage: not given	Sampling: purposive (individuals identified as most appropriate to answer question with experiences of delusions in past 12 months) Interview location: in individuals own homes	Consider the meaning of delusions that occur as part of Parkinson's disease

Tolson et al. (2002) [59]	Individual interviews, supported by diaries, books poems, and newsletters	19 women with Parkinson's disease Mean age: 47 years (range, 34–56 years) Mean duration of illness: 10.0 ± 4.5 years (range, 3–19 years) Hoehn and Yahr stage: not given	Sampling: purposive (women with gynaecological problems) Interview location: study centre	Consider how women adjust to womanhood following Parkinson's and experience and cope with menstruation and gynaecological problems

van der Eijk et al. (2011) [60]	Focus groups discussions	60 individuals 40 (30 = ♂) with Parkinson's disease, 20 (5 = ♂) carers Patients with Parkinson's mean age: 70.5 ± 5.6 years (range, not given) Carers mean age: 63.0 ± 1.8 years (range, not given) Mean duration of illness: 6 ± 5 years (range, not given) Hoehn and Yahr stage: all within stages 1–3.	Sampling: convenience (identified by online web request and from six hospitals) Interview location: not clear	Consider the quality of care received by patients with Parkinson's disease and their carers

Whitehead (2010) [61]	Semistructured interview	8 individuals 4 (3 = ♂) with Parkinson's disease, 4 (1 = ♂) carers Patients with Parkinson's mean age: 64.3 years (range, not given) Carers mean age: 61.0 years (range, not given) Duration of illness: all >5 years (range, not given)	Sampling: purposive (identified from local Parkinson's disease society website) Interview location: not clear	Explore the perspectives of individuals with Parkinson's disease and their spouses on the difficulty with communication

**Table 2:** The summary of results of the COREQ (Tong et al., 2007 [22]) appraisal for the 37 included studies.

Author/year of publication	Domain 1 (8) research team and reflexivity	Domain 2 (15) study design	Domain 3 (9) analysis and findings	Total (32)
Anderson and Fagerlund (2013) [27]	7	9	3	19
Andersson and Sidenvall (2001) [28]	6	11	3	20
Banks and Lawrence (2006) [29]	5	5	4	14
Bingham and Habermann (2006) [30]	6	6	6	18
Bramley and Eatough (2005) [31]	1	10	6	17
Birgersson and Edberg (2004) [32]	4	10	6	20
Buetow et al. (2012) [33]	7	7	6	20
Caap-Ahlgren et al. (2002) [34]	7	10	4	21
Charlton and Barrow (2002) [35]	3	9	5	17
Davis et al. (2003) [36]	7	10	6	23
Delaney et al. (2012) [37]	7	6	5	18
Den Oudsten et al. (2011) [38]	3	8	6	17
Drey et al. (2012) [39]	8	12	7	27
Elliott and Velde (2005) [40]	8	9	5	22
Fleming et al. (2004) [41]	5	8	5	18
Haahr et al. (2011) [42]	7	9	6	22
Habermann (1996) [43]	7	8	5	20
Habermann (1999) [44]	7	7	2	16
Hermanns (2011) [45]	7	9	5	21
Hodgson et al. (2004) [46]	8	13	6	27
Hurt et al. (2012) [47]	5	7	6	18
Jones et al. (2008) [48]	5	10	7	22
Liao et al. (2013) [49]	7	9	6	22
Lindgren (1996) [50]	7	8	6	21
Marr (1991) [51]	7	8	6	21
Miller et al. (2006) [52]	7	8	5	20
Mshana et al. (2011) [53]	5	8	5	18
Oehlberg et al. (2008) [54]	5	6	7	18
Olsson et al. (2013) [55]	6	9	5	20
Soleimani et al. (2014) [56]	6	9	7	22
Stanley-Hermanns and Engebretson (2010) [57]	8	12	8	28
Todd et al. (2010) [58]	3	9	7	19
Tolson et al. (2002) [59]	6	9	4	19
van der Eijk et al. (2011) [60]	2	6	7	15
Whitehead (2010) [61]	6	9	5	20
Benharoch and Wiseman (2004) [62]	8	8	6	20
Pretzer-Aboff et al. (2009) [63]	6	12	7	25
