# Supplementary material for: The Experience of Parkinson's Disease: A Systematic Review and Meta-Ethnography
Source: ScientificWorldJournal. 2014 Nov 30;2014:613592. doi: 10.1155/2014/613592 (PMC4265687; doi:10.1155/2014/613592)
Supplement: Supplementary file 1 — Supplementary File A: Details of the 62 excluded articles. Supplementary File B: The tables illustrating the full thematic analysis. [file 613592.f1.docx]

**Supplementary File A**

Details of the 62 excluded articles

**Conference Proceedings**

1. Lawrence, K. (2013). Failure to manage chronic constipation in community dwelling older people who have Parkinson's disease (PD). Journal of the Australasian Rehabilitation Nurses' Association, 16:12.
2. Thordardottir, B. (2013). Learning experience from a family intervention research project: Benefits for couples living with Parkinson's disease. Journal of Parkinson's disease 3rd World Parkinson Congress Montreal, QC Canada. Conference Start: 20131001 Conference End: 20131004. Conference Publication: (var.pagings). 3 (pp 157-158), 2013. Date of Publication: 2013.
3. Lepage, C., [Legault, C.](http://ovidsp.tx.ovid.com/sp-3.12.0b/ovidweb.cgi?&S=KCDNFPNBPKDDFGDDNCMKDBOBAFCPAA00&Search+Link=%22Legault+C%22.au.). [Lavoie, V.](http://ovidsp.tx.ovid.com/sp-3.12.0b/ovidweb.cgi?&S=KCDNFPNBPKDDFGDDNCMKDBOBAFCPAA00&Search+Link=%22Lavoie+V%22.au.), [L'Ecuyer, N.](http://ovidsp.tx.ovid.com/sp-3.12.0b/ovidweb.cgi?&S=KCDNFPNBPKDDFGDDNCMKDBOBAFCPAA00&Search+Link=%22L%27Ecuyer+N%22.au.), [Lafleur-Prudhomme, E.](http://ovidsp.tx.ovid.com/sp-3.12.0b/ovidweb.cgi?&S=KCDNFPNBPKDDFGDDNCMKDBOBAFCPAA00&Search+Link=%22Lafleur-Prudhomme+E%22.au.), [Pham, M.](http://ovidsp.tx.ovid.com/sp-3.12.0b/ovidweb.cgi?&S=KCDNFPNBPKDDFGDDNCMKDBOBAFCPAA00&Search+Link=%22Pham+M%22.au.), [Emond, S.](http://ovidsp.tx.ovid.com/sp-3.12.0b/ovidweb.cgi?&S=KCDNFPNBPKDDFGDDNCMKDBOBAFCPAA00&Search+Link=%22Emond+S%22.au.), [Dubuc-Boutin, C.](http://ovidsp.tx.ovid.com/sp-3.12.0b/ovidweb.cgi?&S=KCDNFPNBPKDDFGDDNCMKDBOBAFCPAA00&Search+Link=%22Dubuc-Boutin+C%22.au.), & Beland, M.(2013). The question of long-term compliance to the recommendations made by a specialized interdisciplinary team. Journal of Parkinson's Disease. Conference: 3rd World Parkinson Congress Montreal, QC Canada. Conference Start: 20131001 Conference End: 20131004. Conference Publication: (var.pagings). 3 (pp 149-150), 2013. Date of Publication: 2013.
4. [McClurg, D.](http://ovidsp.tx.ovid.com/sp-3.12.0b/ovidweb.cgi?&S=KCDNFPNBPKDDFGDDNCMKDBOBAFCPAA00&Search+Link=%22McClurg+D%22.au.), [Hagen, S.](http://ovidsp.tx.ovid.com/sp-3.12.0b/ovidweb.cgi?&S=KCDNFPNBPKDDFGDDNCMKDBOBAFCPAA00&Search+Link=%22Hagen+S%22.au.), [Cunnington, A. L.](http://ovidsp.tx.ovid.com/sp-3.12.0b/ovidweb.cgi?&S=KCDNFPNBPKDDFGDDNCMKDBOBAFCPAA00&Search+Link=%22Cunnington+AL%22.au.), [Paul, L.](http://ovidsp.tx.ovid.com/sp-3.12.0b/ovidweb.cgi?&S=KCDNFPNBPKDDFGDDNCMKDBOBAFCPAA00&Search+Link=%22Paul+L%22.au.), [Dickinson, L.](http://ovidsp.tx.ovid.com/sp-3.12.0b/ovidweb.cgi?&S=KCDNFPNBPKDDFGDDNCMKDBOBAFCPAA00&Search+Link=%22Dickinson+L%22.au.), [Jamieson, K.](http://ovidsp.tx.ovid.com/sp-3.12.0b/ovidweb.cgi?&S=KCDNFPNBPKDDFGDDNCMKDBOBAFCPAA00&Search+Link=%22Jamieson+K%22.au.), [Aitchison P.](http://ovidsp.tx.ovid.com/sp-3.12.0b/ovidweb.cgi?&S=KCDNFPNBPKDDFGDDNCMKDBOBAFCPAA00&Search+Link=%22Aitchison+P%22.au.) (2013). A qualitative study on the effect of constipation in patients with parkinson's. Neurourology and Urodynamics. Conference: 43rd Annual Meeting of the International Continence Society, ICS 2013 Barcelona Spain. Conference Start: 20130826 Conference End: 20130830. Conference Publication: (var.pagings). 32 (6) (pp 852-853), 2013. Date of Publication: August 2013
5. Tickle-Degnen,  L., Thomas,  C., Saint-Hilaire,  M., Naumova,  E., Ambady,  N., Ellis,  T., Wagenaar,  R. (2012). The social self-management of Parkinson's disease (PD) in daily life. Disease and Movement Disorders, Volume 27,
   June 2012 Abstract Supplement. MDS Abstract of the 16th International Congress of Parkinson's. movement Disorders 2012. Dublin. June 17-21 2012.
6. [Nijhuis F.A.P.](http://ovidsp.tx.ovid.com/sp-3.12.0b/ovidweb.cgi?&S=KCDNFPNBPKDDFGDDNCMKDBOBAFCPAA00&Search+Link=%22Nijhuis+FAP%22.au.) [Van Heek J.](http://ovidsp.tx.ovid.com/sp-3.12.0b/ovidweb.cgi?&S=KCDNFPNBPKDDFGDDNCMKDBOBAFCPAA00&Search+Link=%22Van+Heek+J%22.au.) [Faber M.J.](http://ovidsp.tx.ovid.com/sp-3.12.0b/ovidweb.cgi?&S=KCDNFPNBPKDDFGDDNCMKDBOBAFCPAA00&Search+Link=%22Faber+MJ%22.au.) [Post B.](http://ovidsp.tx.ovid.com/sp-3.12.0b/ovidweb.cgi?&S=KCDNFPNBPKDDFGDDNCMKDBOBAFCPAA00&Search+Link=%22Post+B%22.au.) [Bloem B.R.](http://ovidsp.tx.ovid.com/sp-3.12.0b/ovidweb.cgi?&S=KCDNFPNBPKDDFGDDNCMKDBOBAFCPAA00&Search+Link=%22Bloem+BR%22.au.) (2012). Shared decision making in advanced Parkinson's disease: Current clinical practice and the way forward. Movement Disorders. Conference: 16th International Congress of Parkinson's Disease and Movement Disorders Dublin Ireland. Conference Start: 20120617 Conference End: 20120621. Conference Publication: (var.pagings). 27 (pp S309), 2012. Date of Publication: June 2012
7. [Nitsch R.M.](http://ovidsp.tx.ovid.com/sp-3.12.0b/ovidweb.cgi?&S=KCDNFPNBPKDDFGDDNCMKDBOBAFCPAA00&Search+Link=%22Nitsch+RM%22.ed.) [Fisher A.](http://ovidsp.tx.ovid.com/sp-3.12.0b/ovidweb.cgi?&S=KCDNFPNBPKDDFGDDNCMKDBOBAFCPAA00&Search+Link=%22Fisher+A%22.ed.) [Windisch M.](http://ovidsp.tx.ovid.com/sp-3.12.0b/ovidweb.cgi?&S=KCDNFPNBPKDDFGDDNCMKDBOBAFCPAA00&Search+Link=%22Windisch+M%22.ed.) [Hanin I.](http://ovidsp.tx.ovid.com/sp-3.12.0b/ovidweb.cgi?&S=KCDNFPNBPKDDFGDDNCMKDBOBAFCPAA00&Search+Link=%22Hanin+I%22.ed.) (2011). Junior doctors experiences of managing inpatients with parkinson's disease: A qualitative study. Neurodegenerative Diseases. Conference: 10th International Conference AD/PD - Alzheimer's and Parkinson's Diseases: Advances, Concepts and New Challenges Barcelona Spain. Conference Start: 20110309 Conference End: 20110313. Conference Publication: (var.pagings). 8 , 2011. Date of Publication: March 2011.
8. [Connolly D.](http://ovidsp.tx.ovid.com/sp-3.12.0b/ovidweb.cgi?&S=KCDNFPNBPKDDFGDDNCMKDBOBAFCPAA00&Search+Link=%22Connolly+D%22.au.) [Cassidy C.](http://ovidsp.tx.ovid.com/sp-3.12.0b/ovidweb.cgi?&S=KCDNFPNBPKDDFGDDNCMKDBOBAFCPAA00&Search+Link=%22Cassidy+C%22.au.) [Mhurchu E.N.](http://ovidsp.tx.ovid.com/sp-3.12.0b/ovidweb.cgi?&S=KCDNFPNBPKDDFGDDNCMKDBOBAFCPAA00&Search+Link=%22Mhurchu+EN%22.au.) [Nolan E.](http://ovidsp.tx.ovid.com/sp-3.12.0b/ovidweb.cgi?&S=KCDNFPNBPKDDFGDDNCMKDBOBAFCPAA00&Search+Link=%22Nolan+E%22.au.) (2010). An exploration of individuals' perspectives on the impact of Parkinson's Disease on self-care, work, and leisure activities.
   Movement Disorders. Conference: 2nd World Parkinson Congress, WPC Glasgow United Kingdom. Conference Start: 20100928 Conference End: 20101001. Conference Publication: (var.pagings). 25 (pp S712), 2010. Date of Publication: 15 Sep 2010.
9. [Barretto M.](http://ovidsp.tx.ovid.com/sp-3.12.0b/ovidweb.cgi?&S=KCDNFPNBPKDDFGDDNCMKDBOBAFCPAA00&Search+Link=%22Barretto+M%22.au.) [Singhal B.S.](http://ovidsp.tx.ovid.com/sp-3.12.0b/ovidweb.cgi?&S=KCDNFPNBPKDDFGDDNCMKDBOBAFCPAA00&Search+Link=%22Singhal+BS%22.au.) [Lalkaka J.](http://ovidsp.tx.ovid.com/sp-3.12.0b/ovidweb.cgi?&S=KCDNFPNBPKDDFGDDNCMKDBOBAFCPAA00&Search+Link=%22Lalkaka+J%22.au.) [Wadia P.](http://ovidsp.tx.ovid.com/sp-3.12.0b/ovidweb.cgi?&S=KCDNFPNBPKDDFGDDNCMKDBOBAFCPAA00&Search+Link=%22Wadia+P%22.au.) [Devare S.](http://ovidsp.tx.ovid.com/sp-3.12.0b/ovidweb.cgi?&S=KCDNFPNBPKDDFGDDNCMKDBOBAFCPAA00&Search+Link=%22Devare+S%22.au.) (2010). An analysis of the needs of homebound patients with Parkinson's disease and their caregivers in the Indian subcontinent - A qualitative study. Movement Disorders. Conference: 2nd World Parkinson Congress, WPC Glasgow United Kingdom. Conference Start: 20100928 Conference End: 20101001. Conference Publication: (var.pagings). 25 (pp S710-S711), 2010. Date of Publication: 15 Sep 2010.
10. [Beaudet L.](http://ovidsp.tx.ovid.com/sp-3.12.0b/ovidweb.cgi?&S=KCDNFPNBPKDDFGDDNCMKDBOBAFCPAA00&Search+Link=%22Beaudet+L%22.au.) [Ducharme F.](http://ovidsp.tx.ovid.com/sp-3.12.0b/ovidweb.cgi?&S=KCDNFPNBPKDDFGDDNCMKDBOBAFCPAA00&Search+Link=%22Ducharme+F%22.au.) (2010). Elderly couples in transition: Their experiences at the moderate stage of Parkinson's. Movement Disorders. Conference: 2nd World Parkinson Congress, WPC Glasgow United Kingdom. Conference Start: 20100928 Conference End: 20101001. Conference Publication: (var.pagings). 25 (pp S695-S696), 2010. Date of Publication: 15 Sep 2010
11. [Tremblay C.](http://ovidsp.tx.ovid.com/sp-3.12.0b/ovidweb.cgi?&S=KCDNFPNBPKDDFGDDNCMKDBOBAFCPAA00&Search+Link=%22Tremblay+C%22.au.) [Blais J.](http://ovidsp.tx.ovid.com/sp-3.12.0b/ovidweb.cgi?&S=KCDNFPNBPKDDFGDDNCMKDBOBAFCPAA00&Search+Link=%22Blais+J%22.au.) [Lachance J.](http://ovidsp.tx.ovid.com/sp-3.12.0b/ovidweb.cgi?&S=KCDNFPNBPKDDFGDDNCMKDBOBAFCPAA00&Search+Link=%22Lachance+J%22.au.) [Monchi O.](http://ovidsp.tx.ovid.com/sp-3.12.0b/ovidweb.cgi?&S=KCDNFPNBPKDDFGDDNCMKDBOBAFCPAA00&Search+Link=%22Monchi+O%22.au.) [Monetta L.](http://ovidsp.tx.ovid.com/sp-3.12.0b/ovidweb.cgi?&S=KCDNFPNBPKDDFGDDNCMKDBOBAFCPAA00&Search+Link=%22Monetta+L%22.au.)(2010) Time-course and qualitative analysis of unconstrained oral naming abilities in non-demented Parkinson's individuals. Movement Disorders. Conference: 14th International Congress of Parkinson's Disease and Movement Disorders Buenos Aires Argentina. Conference Start: 20100613 Conference End: 20100617. Conference Publication: (var.pagings). 25 (pp S328-S329), 2010. Date of Publication: 2010.
12. [McRae C.](http://ovidsp.tx.ovid.com/sp-3.12.0b/ovidweb.cgi?&S=KCDNFPNBPKDDFGDDNCMKDBOBAFCPAA00&Search+Link=%22McRae+C%22.au.) [Ene H.](http://ovidsp.tx.ovid.com/sp-3.12.0b/ovidweb.cgi?&S=KCDNFPNBPKDDFGDDNCMKDBOBAFCPAA00&Search+Link=%22Ene+H%22.au.) [Pinarowicz J.](http://ovidsp.tx.ovid.com/sp-3.12.0b/ovidweb.cgi?&S=KCDNFPNBPKDDFGDDNCMKDBOBAFCPAA00&Search+Link=%22Pinarowicz+J%22.au.) [Schenkman M.](http://ovidsp.tx.ovid.com/sp-3.12.0b/ovidweb.cgi?&S=KCDNFPNBPKDDFGDDNCMKDBOBAFCPAA00&Search+Link=%22Schenkman+M%22.au.) (2009). Motivation to exercise: Qualitative reflections on a clinical trial of three exercise interventions for persons with parkinson's disease. Movement Disorders. Conference: 23rd Annual Symposium on Etiology, Pathogenesis, and Treatment of Parkinson's Disease and Other Movement Disorders Baltimore, MD United States. Conference Start: 20091011 Conference End: 20091011. Conference Publication: (var.pagings). 24 (12) (pp 1879-1880), 2009. Date of Publication: 15 Sep 2009
13. Cicely, K., Howeard, C. E., Lloyd, E., Cooper, J. A., Johnston, K., McLntosh, E., Lloyd, A. J. (2013). Living with Parkinson’s: impact and importance of ‘on’ and ‘off’ time. 3^rd^ World Parkinsons Congress. Palais de Congres, Montreal, Canada, 1-4. 2013.

**Different Language**

1. García, J. M., Solís, M. J. (2005). Nurses who treat Parkinson patients: what do they know and what do they want to know? Revista de Enfermería 28: 42-50
2. Dressen, C., Brandel, J. P., Schneider, A., Magar, T., Rnon, D., Ziēgler, M. (2007). Effects of Parkinson’s disease on quality of life patients’ spouses: a qualitative survey. Revue Neruolgoique, 163: 801-807.
3. Navarro-Peternella, F. M., Marcon, S. S. (2010). Living with Parkinson's disease from the perspective of parkinsonians and their relatives. Revista Gaucha de Enfermagem. 31(3):415-22
4. Lubi, K., Vihalemm, T., Taba, P.(2014). Patsiendid infootsijana: valjakutsed, voimalused ja ohud Parkinsoni tovega patsientide naitel. Eesti Arst, 93: 76-82
5. Haapaniemi, H., Leino, P., Routasalo, P. (2005).Parkinson's disease patients' and their carers' experiences at different stages of the disease. Hoitotiede, 17: 311-322.
6. [Portillo Vega M.C.](http://ovidsp.tx.ovid.com/sp-3.12.0b/ovidweb.cgi?&S=KCDNFPNBPKDDFGDDNCMKDBOBAFCPAA00&Search+Link=%22Portillo+Vega+MC%22.au.) [Senosiain Garcia J.M.](http://ovidsp.tx.ovid.com/sp-3.12.0b/ovidweb.cgi?&S=KCDNFPNBPKDDFGDDNCMKDBOBAFCPAA00&Search+Link=%22Senosiain+Garcia+JM%22.au.) [Arantzamendi Solabarrieta M.](http://ovidsp.tx.ovid.com/sp-3.12.0b/ovidweb.cgi?&S=KCDNFPNBPKDDFGDDNCMKDBOBAFCPAA00&Search+Link=%22Arantzamendi+Solabarrieta+M%22.au.) [Zaragoza Salcedo A.](http://ovidsp.tx.ovid.com/sp-3.12.0b/ovidweb.cgi?&S=KCDNFPNBPKDDFGDDNCMKDBOBAFCPAA00&Search+Link=%22Zaragoza+Salcedo+A%22.au.) [Navarta Sanchez M.V.](http://ovidsp.tx.ovid.com/sp-3.12.0b/ovidweb.cgi?&S=KCDNFPNBPKDDFGDDNCMKDBOBAFCPAA00&Search+Link=%22Navarta+Sanchez+MV%22.au.) [De Cerio Ayesa S.D.](http://ovidsp.tx.ovid.com/sp-3.12.0b/ovidweb.cgi?&S=KCDNFPNBPKDDFGDDNCMKDBOBAFCPAA00&Search+Link=%22De+Cerio+Ayesa+SD%22.au.) [Riverol Fernandez M.](http://ovidsp.tx.ovid.com/sp-3.12.0b/ovidweb.cgi?&S=KCDNFPNBPKDDFGDDNCMKDBOBAFCPAA00&Search+Link=%22Riverol+Fernandez+M%22.au.) [Martinez Vila E.](http://ovidsp.tx.ovid.com/sp-3.12.0b/ovidweb.cgi?&S=KCDNFPNBPKDDFGDDNCMKDBOBAFCPAA00&Search+Link=%22Martinez+Vila+E%22.au.) [Luquin Piudo M.R.](http://ovidsp.tx.ovid.com/sp-3.12.0b/ovidweb.cgi?&S=KCDNFPNBPKDDFGDDNCMKDBOBAFCPAA00&Search+Link=%22Luquin+Piudo+MR%22.au.) [Ursua Sesma M.E.](http://ovidsp.tx.ovid.com/sp-3.12.0b/ovidweb.cgi?&S=KCDNFPNBPKDDFGDDNCMKDBOBAFCPAA00&Search+Link=%22Ursua+Sesma+ME%22.au.) [Corchon Arreche S.](http://ovidsp.tx.ovid.com/sp-3.12.0b/ovidweb.cgi?&S=KCDNFPNBPKDDFGDDNCMKDBOBAFCPAA00&Search+Link=%22Corchon+Arreche+S%22.au.) [Moreno Lorente V.](http://ovidsp.tx.ovid.com/sp-3.12.0b/ovidweb.cgi?&S=KCDNFPNBPKDDFGDDNCMKDBOBAFCPAA00&Search+Link=%22Moreno+Lorente+V%22.au.) (2012). Patients and relatives living with Parkinson's disease: Preliminary results of Phase i. Revista Cientifica de la Sociedad Espanola de Enfermeria Neurologica, 36:31-38
7. [Navarro-Peternella F.M.](http://ovidsp.tx.ovid.com/sp-3.12.0b/ovidweb.cgi?&S=KCDNFPNBPKDDFGDDNCMKDBOBAFCPAA00&Search+Link=%22Navarro-Peternella+FM%22.au.) [Marcon S.S.](http://ovidsp.tx.ovid.com/sp-3.12.0b/ovidweb.cgi?&S=KCDNFPNBPKDDFGDDNCMKDBOBAFCPAA00&Search+Link=%22Marcon+SS%22.au.) (2010). Living with Parkinson's disease from the perspective of parkinsonians and their relatives. Revista gaucha de enfermagem / EENFUFRGS, 31: 415-422.
   [Peternella F.M.](http://ovidsp.tx.ovid.com/sp-3.12.0b/ovidweb.cgi?&S=KCDNFPNBPKDDFGDDNCMKDBOBAFCPAA00&Search+Link=%22Peternella+FM%22.au.) [Marcon S.S.](http://ovidsp.tx.ovid.com/sp-3.12.0b/ovidweb.cgi?&S=KCDNFPNBPKDDFGDDNCMKDBOBAFCPAA00&Search+Link=%22Marcon+SS%22.au.) (2009). Diagnosed with Parkinson's diesease: impact on patients and family members. Revista brasileira de enfermagem, 62: 25-31
8. Busse, M. (2006). What Parkinson effects in persons: the experience of Parkinson's disease and its relevance for nursing [German]. PR-Internet fur die Pflege (PR INTERNET PFLEGE), 8: 362-70.
9. Salcedo, A. Z., García, J. M. S., Fernández, M. R., Bravo, S. A., de Cerio Ayesa, S. D., Sesma, M. E. U., Portillo, M. U. (2014). *Key elements in the process of living with Parkinson’s disease for patients and caregivers. An. Sist. Sanit. Navar, 37: 69-80.*

**Reported on the Experience of a Programme or Intervention**

1. Crizzle, A. M., Newhouse, I. J. (2012). Themes associated with exercise adherence in persons with Parkinson's disease: a qualitative study. Occupational Therapy in Health Care, 2-3: 174-186.
2. Haahr, A., Kirkevold, M., Hall, E. O. C., Østergaard, K. (2010). From miracle to reconciliation: A hermeneutic phenomenological study exploring the experience of living with Parkinson’s disease following Deep Brain Stimulation Focus on Caregivers or others. International Journal of Nursing studies, 47: 1228-1236.
3. Hirsch, M. A., Sanjak, M., Englert, D., Lyer, S., Quinlan, M. M. (2014). Parkinson patients as partners in care. Parkinsonism and Related Disorders, S174-S179.
4. Quinn, L., Busse, M., Khalil, H., Richardons, S., Rosser, A., Morris, H. (2010). Client and therapist views on exercise programmes for early-mid stage Parkinson’s disease and Huntington’s disease. Disability and Rehabilitation, 32: 917-928.

**Focus on Care Givers**

1. Berry, R. A., Murphy, J. F. (1995). Well-being of caregivers of spouses with Parkinson’s Disease. Clinical Nursing Research, 4, 373-386.
2. Chambers, M., Ryan, A. A. (2001). Exploring the emotional support needs and coping strategies of family carers. Journal of Psychiatric and Mental Health Nursing, 8: 99-106.
3. Davey, C., Wiles, R., Ashburn, A., Murphy, C. (2004). Falling in Parkinson’s: the impact on informal caregivers. Disability and Rehabilitation, 26: 1360-1366.
4. Eriksson, M., Svedlund, M. (2006). ‘The intruder’: spouses’ narratives about life with a chronically ill partner. Journal of Clinical Nursing, 15: 324-333.
5. Haahr, A., Kirkevold, M., Hall, E. O. C., Østergaard, K. (2013). ‘Being in it together’: living with a partner receiving deep brain stimulation for advanced Parkinson’s disease – a hermeneutic phenomenological study. Journal of Advanced Nursing, 69: 338-347.
6. Pasetti, C., Ferrario, S. R., Fornara, R., Picco, D., Foglia, C., Galli, J. (2003). Caregiving and Parkinson’s disease. Neurological Science, 24: 203-204.
7. Habermann, B. (2000). Spousal perspective of Parkinson’s disease in middle life. Journal of Advanced nursing, 34: 1409-1415.
8. McLaughlin, D., Hasson, F., Kernohan, W. G., Waldron, M., McLaughlin, M., Cochrane, B., Chambers, H. (2011). Living and coping with Parkinson’s disease: Perceptions of informal carers. Palliative Medicine, 25: 177-182
9. McRae, C., Sherry, P., Roper, K. (1999). Stress and family functioning among caregivers of persons with Parkinson’s disease. Parkinsonism and Related Disorders, 5: 69-75.
10. Tan, S. B., Williams, A. F., Morris, M. E. (2012). Experiences of caregivers of people with Parkinson’s disease in Singapore: a qualitative analysis. Journal of Clinical Nursing, 21: 2235-2246.

**Mixed Sample**

1. [Baylor](http://ajslp.pubs.asha.org/solr/searchResults.aspx?author=Carolyn+Baylor), C., Burns, M.,Eadie, T., Britton, D., Yorkston, K. (2011). A Qualitative Study of Interference With Communicative Participation Across Communication Disorders in Adults. American Journal of Speech-Language Pathology November, 20: 269-287
2. Frazier, L. D., Cotrell, V., Hooker, K. (2003). Possible selves and illness: A comparison of individuals with Parkinson’s Disease, early-stage Alzheimer’s disease, and healthy older adults. International Journal of Behavioural Development, 27: 1-11
3. Hartley, S., McArthur, M., Coenen, M., Cabello, M., Covelli, V., Roszczynska-Michta, J., Pitkanen, T., Bickenbach, J., Cieza, A. (2014). Narratives Reflecting the Lived Experiences of People with Brain Disorders: Common Psychosocial Difficulties and Determinants. Plosone, 9:5: e96890.
4. Kitchen, R., Shirlow, P., Shuttleworth, I. (1998). On the Margins: disabled people’s experience of employment in Dongegal, West Ireland. Disability and Society, 13: 785-806
5. Hermann, M., Freyholdt, U., Fuch, G., & Wallesch, C. W. (1997). Coping with chronic neurological impairment: A contrastive analysis of Parkinson’s disease and stroke. Disability and Rehabilitation, 19, 6–12.
6. Hainsworth, M. A., Eakes, C. G., Burke, M. L. (1994). Coping with Chronic Sorrow. Issues in Mental Health Nursing, 15: 59-66.
7. Kullberg, K., Bjorklund, A., Sidenvall, B., Aberg, A. C. (2011). ‘I start my day by thinking about what we’re going to have for dinner’ – a qualitative study on approaches to food-related activities among elderly men with somatic diseases. Scandinavian Journal of Caring Sciences, 25: 227-234.
8. Mazanderani, F., Locock, L., Powell, J. (2012). Being differently the same: The mediation of identity tensions in the sharing of illness experiences. Social Science and Medicine, 74:546-553.
9. MacCabe, M. P., O’Connor, E. J. (2012). Why are some people with neurological illness more resilient than others? Psychology, Health and Medicine, 17: 17-34.

**Other type of Design or Article**

1. Abudi, S., Bar-Tal, Y., Ziv, L., Fish, M. (1997). Parkinson’s disease symptoms – patients’ perceptions. Journal of Advanced Nursing, 25: 54-59.
2. Brod, M., Mendelsohn, G. A., Roberts, B. (1998). Patients’ Experiences of Parkinson’s Disease. Journal of Gerontology, 53: 213-222.
3. Zakzanis, K. K., Freedman, M. (1999). A neuropsychological comparison of demented and nondemented patients with Parkinson's disease. Applied Neuropsychology, 6:129-146.
4. Wielinski, C. L., Varpness, S. C., Erickson-Davis, C., Paraschos, A. J., Parashos, S. A. (2009). Quantitative study: Sexual and Relationship Satisfaction among Persons with Young-Onset Parkinson's Disease. Journal of Sexual Medicine, 7: 1438-1444.
5. Habermann, B., Hines, D., Davis, L. (2013). Caring for parents with neurodegenerative disease: a qualitative description. Clinical Nurse Specialist, 27: 181-187.
6. Brown, R. G., Jahanshahi, M., Quinn, N., Marsden, C. D. (1990). Sexual function in patients with Parkinson’s disease and their partners. Journal of Neurology, Neurosurgery, and Psychiatry, 53: 480-486.
7. Caap-Ahlgren, M., Dehlin, O., (2001). Insomnia and depressive symptoms in patients with Parkinson’s disease. Relationship to health-related quality of life. An interview study of patients living at home. Achieves of Gerontology and Geriatrics, 32: 23-33.
8. Cleary, R. A., Poliakoff, E., Galpin, A., Dick, J. R. R., Holler, J. (2011). An investigation of co-speech gesture production during action description in Parkinson’s disease. Parkinsonism and Related Disorders, 17: 753-756.
9. Fine, E. M., Delis, D. C., Paul, B. M., Filoteo, V. (2011) Reduced verbal fluency for proper names in nondemented patients with Parkinson's disease: A quantitative and qualitative analysis, Journal of Clinical and Experimental Neuropsychology, 33:2, 226-233
10. Goebel, S., Atanassov, L., Kohnken, G., Mehdorn, H. M., Leplow, B. (2013). Understanding quantitative and qualitative figural fluency in patients with Parkinson’s disease. Neurological Science, 34: 1383-1390.
11. Gotham, A-M., Brown, R. G., Marsden, C. D. (1986). Depression in Parkinson’s disease: a quantitative and qualitative analysis. Journal of Neurology, Neurosurgery, and Psychiatry, 49: 381-389.
12. Haeske-Dewick, h. C. (1996). Are perception and memory for faces influenced by a specific age at onset factor in Parkinson’s disease? Neuropsycholgia, 34: 315-320.
13. Konstam, V., Holmes, W., Wilczenski, F., Baliga, S., Lester, J., Priest, R. (2003). Meaning in the lives of caregivers of individuals with Parkinson’s disease. Journal of Clinical Psychology in Medical Settings, 10: 17-25
14. Posen, J., Moore, O., Tassa, D. S., Ginzburg, K., Drory, M., Giladi, N. (2001) Young Women with PD. Social Work in Health Care, 32:1, 77-91

**Supplementary File B**

The tables illustrating the full thematic analysis.

Table 1 The effects of Parkinson’s disease on an individual’s identity and self

| **Sub-Theme** | **Code** | **Unit** | **Studies** |
| --- | --- | --- | --- |
| Effects of illness on identity and sense of self | Core sense of self unchanged | Individuals highlighted different ways in which aspects considered central to their self and identity were continued or retained. Those who knew the individuals well could perhaps have a greater understanding that person had not changed. Individuals identities that did not change included their identity within a family, within a job and the meaningful activities that were associated with this identity. For instance one individual stated “I don’t find things uncomfortable with my family, erm nor Mark [husband], nor Charles [bereaved husband], I didn’t find things uncomfortable, because they know who I am inside.” [5]. Individuals could also acknowledge the Parkinson’s whilst identifying that they had not changed apart from this for instance, “I am still the person I was with one minor adjustment; I have Parkinson’s disease and “I just want people to see me” were common.” [31]. This comment highlights the desire to be seen as a person rather than a disease, thus empathising a certain ability to retain or continue unchanged. | 5,12,14,17,18,31 |
|  | Retaining identity | Individuals could identify the importance of retaining their old identities, this can be observed also in retaining normalcy as well as consideration by individuals that the core self was unchanged and the emphasis of being resilient to the Parkinson’s. Some individuals distanced themselves to the identity of a patient and empathised positive identity and attitudes, for example, one stated “I don’t consider myself a patient yet. When I’m a patient, I’m horizontal in a bed and that’s it. But until then, I’m pretty active.” (NM2) [9]. Individuals were able to continue certain roles like jobs, with considerations and aids to maintain important identities. For instance, one individual stated “I am still able to work, my employer has made some arrangements for me”. [12]. Other individuals empathised that they could still perform roles well for instance being a ‘good’ mother to their children. Individuals also identified that maintaining important activities like going out to dinner help the process of continuing their previous identity. The ability to retain identity could be a challenging task as illustrated by one individual, ”Yesterday I couldn’t get turned on. I went to work with the pump on. But I couldn’t get on. I couldn’t get enough medication. It just wasn’t working. I was shaking, I had a terrible tremor...So I have to learn how to adjust this when I’m under pressure. I have to figure out how it works with stress and anxiety [17] | 9,10,12,15,16,17,18,21,32 |
|  | The influence of the illness identity and the self seen as the disease symptoms | The disease of Parkinson’s and the symptoms meant individual could feel trapped in a body that didn’t represent who they wanted to be. Stereotypical characteristics of the disease and symptoms could induce a sense of shame and incompetence, be seen as representing a label of them, for instance one individual stated “I didn’t really want to admit that I was one of them, the disabled”[9]. Alternatively some individuals distinguished between what their body represented and what their mind wanted, with the prevention of movement, skills or abilities being affected individuals would have to, to some extent, consider the association with Parkinson’s as part of their identity. | 8,9,12,17,18,20,25,31 |
|  | The loss and alteration of self and important identities and roles  Previous self  General identity e.g., as a women  Linked with social perception  -linked to imaging  -linked to autonomy | Individuals emphasised a contrast and conflict between how they want to be seen and what their body represents, this contrast was often made in relationship to how they used to be and related to a loss of ability and function in important social roles, roles that they used to obtain. For instance one participant stated, “I used to be so confident, I could get up, get my children ready, clean the house, get them to school, get home again for when they came out of school” [5.]. Importantly, the loss is empathised in social settings if people don’t know the individual or what they are diagnosed with, the effects of the Parkinson’s could be so great that individual could not identify with who they were before for instance, one individual stated “It’s as alien to me now as if it was someone else [ . . . ] It’s murked the water so much that I don’t really know what I was like before, or really have much faith in what I was like before. (P10) [11].  Different losses were evident relating to an individual’s identity, these could be loss to an individual’s identity at work, for instance one individual stated “I probably would have not retired when I did and [would] have stayed on with my company because I enjoy my work”[14].Another individual who had a strong identity in the military contrasted this with his current state, “I wish you could see me the way that I was. I am just worthless and stupid now. I was in the military and had a sharp mind. I could build a lot of things with my hands, now my hands won’t work.” [31]. The losses could relate to family life require role changes for instance, in one study a husband had to support the changing of sanitary towels and this resulted in “it put him off anything sexual.”[15]. Alternatively, individuals could not be able to perform traditional roles and tasks as a parent or grandparent, as well as more general roles like that of a women and femininity. The losses felt extended to their plans for the future in addition to contrasting the present with the past. Further to this there was a need to accept the loss of such integral roles for instance, one individual stated *“I realized that I would never be the same.…I could never go back to*  *being the old me, and I am okay with that, now. It took me some time to get to the point that I was okay with it…”[19]* | 3,4,5,8,11,12,14,15,16,17,19,20,23,24,27,29,31,32,35,36 |
| The effect on activities and function | Loss of meaningful activities  *link to loss of roles (combine the two) | Individuals explicitly identified the loss of meaningful activities and interactions that were associated with their identity, for instance the inability to cook and provide for the family, or mending clothes that were damaged, or performing tasks related to personal hygiene. Other meaningful activities and interactions that were missed included the inability to undertake hobbies, holidays or tasks they once enjoyed, the ability to see friends, go to groups and organised activities like church. For instance, one individual stated “I feel like I am locked in confinement…I can’t do things that I want to do,” [31]. It was also hard for some individuals to see others taking on activities that they could do. | 8,12,14,15,16,17,19,21,23,24,25,30,31,32 |
|  | Loss of function  *link to inability to move | Small tasks of daily living could be effected, this included taken for granted aspects of living, such as eating and writing, cooking, or going shopping. When the tasks could be completed they were affected by an individual’s level of fatigue and energy as well as being performed in a slower or modified way. This loss of function was summarised well by one individual, “You can’t do what you normally want to do or like I can’t tie my shoe sometimes. Now I am to the point that I can’t get dressed without help. Everything that I go to do is hard to do. Getting the lids off of jars or opening a box or trying to read or anything I do is more difficult…Things you just take for granted until you can’t do it.” [31]. Another participant identifies the changes in how to negotiate living in their home environment ‘I find it hard to get about’ difficulties negotiating stairs, changing direction and transfers were reported, such as getting out of the bath. [36]. Finally, another individual illustrate how an activity was modified “I can dress my top half, I can do my bra and top but I can’t do the bottom half. I can do one leg but I put the bad leg in first and then I can’t get the good leg in. I just can’t do it. And I cannot wear heeled shoes at all.” [15] | 8,9,10,12,14,15,16,17,19,21,22,23,27,28,31,35,36, |

Note: 1 = Anderson, 2013, 2 = Andersson (2001), 3 = Banks(2006), 4 = Bingham (2006), 5 = Bramley (2014), 6 = Birgersson (2004), 7 = Buetow (2012), 8 = Capp-Ahlgren (2002), 9 = Charlton (2002), 10 = Davis (2004), 11 = Delaney (2012), 12 = Den Oudsten (2011), 13 = Drey (2012) , 14 = Elliott (2005), 15 = Flemming (2004), 16 = Haahr (2010), 17 = Habermann (1996), 18 = Habbermann (1999), 19 = Hermans (2011), 20 = Hodgson (2004) , 21 = Hurt (2012), 22 = Jones (2008), 23 = Liao (2013), 24 = Lindgren (1996), 25 = Marr (1991), 26 = Miller (2006), 27 = Mshana (2011), 28 = Oehlberg (2008), 29 = Olsson (2013), 30 = Soleimani (2014), 31 = Stanley-Hermanns (2010), 32 = todd (2010), 33 = Tolson (2002), 34 = Van Der Eijk (2011), 35 = Whitehead (2010), 36 = Benharoch (2004), 37 = Pretzer-Aboff (2009).

Table 2 The Psychosocial Challenges and Adjustment to Parkinson’s Disease

| **Sub-theme** | **Code** | **Unit** | **Studies** |
| --- | --- | --- | --- |
| Psychosocial challenges | Social confidence, anxiety, esteem and competence and being self-conscious, | A major challenge for individuals was an increasing lack of physical confidence and competence, the loss of social roles (e.g., like not being able to be the grandmother or mother to their children they would like) as well as social confidence and self-esteem (e.g., feeling able to go out in public), as a result of the Parkinson’s disease and symptoms. This was often increased by past experiences such as freezing in public, falling or not being able to communicate in a clear way with others. For instance one individual stated: ‘Embarrassed, people just keep staring at you when you cannot get your words out. . . so I just avoid the people’ (M 82, DD 4 years, HY 3, IM 47). [26]  Feelings when in social situations included feeling conspicuous and being embarrassed of one self for different reasons including, the inability to perform tasks or movement, for instance one individual stated “it’s deplorable the way I eat. They out to put me in a pigpen” [14]. Social anxiety was increase in unknown situations, and crowded situations where individuals would feel more conspicuous and lack confidence existed.  Effects of reduce confidence, included eating less when others were around, not engaging in tasks and activities for safety reasons and general social withdrawal and contact. For instance, one individual stated “I’m quite self-conscious about that, so I don’t go to the theatre now” [36] | 2,3,8,12,14,15,16,18,20,22,23,24,25,26,29,30,33,35,36 |
|  | meta-perceptions and stigma | Individuals were worried about what others thought of them, especially in social or public setting, feelings of stigma were prominent. Individuals could perceive other to be staring at them because of a Parkinson’s related symptoms. For instance one individual stated “Feel especially watched. People glance and wonder what is wrong”[8]. Individuals were sensitive to how others perceived them in other ways for instance; one participant stated `many got embarrassed when she sat in a wheelchair' [2] and “And these boys, about 13, 14 stopped and stared at me and I could tell by the way they were staring and talking that they were saying, ‘He’s drunk’. So … er … that’s the feeling that people who don’t know what’s wrong they make the wrong decisions about you.” [35]  It was identified to be humiliating to suffer an ‘off’ period or symptoms in public, like freezing, shaking, or stumbling and falling. This made them think that others were looking down at them. In a similar way it could be humiliating to be within a conversation if they had a masked face or muffled voice. For instance, one participant stated ”People don’t understand me and think I’m an idiot. In fact, I’m very clear in the head. It’s really an awful feeling” [23]  The result of this was a general sense of negative feelings, negative self-evaluation and social withdrawal. For instance, one participant stated “Even if people in town know that I am slow …you really don’t feel good… I simply don’t want to advertise for the disease.” [16]. For some individuals it could influence how others around them acted for instance one women stated “My daughter wouldn’t really bring her friends round to the house so much, she went out to other people’s houses more. Although she probably wouldn’t say so herself, I feel it was to do with the Parkinson’s.” [15] and another commented “The number one complaint my family has is they said that I always look like I am mad.” [31]. | 2,3,5,6,8,12,13,15,16,20,22,23,25,26,27,29,31,35 |
| Psychological adjustment to the disease | Elevated levels of anxiety and stress and  Inability and difficulty at dealing with stress | Individuals reported that the psychological impact of Parkinson’s included anxiety, social anxiety, panic attacks and stress. These feelings could impact on the ability to undertake activities of daily living, like household duties and sleep.  Individuals reported difficulties at dealing with stressful situations e.g., at a new job interview, in a crowded place, as a result of pain, sorting change for payment of goods, holding a song book at church. Various physiological responses to stress including; trembling as a result of stress, this was identified within individuals hands, having their foot stick to the floor, perceiving the brain slowing down and affecting communication. Some causes were mentioned like having a crowed environment or when they felt tired. For instance, “It’s the tiredness; it’s the memory. You can’t get your thoughts together; you can’t plan. You get frustrated. It’s like becoming demented. It’s like, I can’t find the right words.” [29] | 1,2,3,5,8,13,14, 29,36 |
|  | Frustration | Frustration was an extremely common emotion reported consistently across studies. The reason for it included the inability to engage in physical activities, activities of daily leaving, being dependant on others for support of basic or mundane tasks, as well as lack of energy to undertake activities. For instance one individual stated: ‘I get frustrated with not being as mobile as I would like.’ [36] and another stated *it is just the sheer frustration that you can’t do anything. I live a life of frustration [19]*. Frustration was also associated with anger, for instance one individual stated *“If I suddenly get a moment when I think about it I get angry but not every day, just occasionally.” [21]* | 8,15,19,21,25,28,29,31,33,35,36,37 |
|  | Loss of control and uncertainty in the present and future  -note having a sense of purpose provides a sense of normalcy, control and purpose  -process? acknowledging something wrong, acknowledging illness, accepting illness  *barrier against autonomy  *link to negative imaging | Control was taken by Parkinson’s at the symptoms exerted control of individual’s life, functioning and ability to interact. Control was lost in respect to physical tasks like driving a car, specific roles like being a father or the ability to regain a sense of purpose. Control was removed by the reliance on medication, symptoms increasing and causing uncertainty about symptoms, fluctuations in functioning and body movements. Falls for example were very possible one participant stated “I was in the parking lot and. All of a sudden, a tremendous force like propelled me, and I was going really fast, and fortunately I was able to put my hands up” [10].  Individuals highlighted the lack of control of planning because of an inability to trust the disease, for instance one individual stated “if I only knew that I could trust time. Then it is no trouble. But I get talking to people and forget everything about those rotten pills. And suddenly… I am stuck. [16]. As a consequence individuals would opt for more controllable situations and placed.  The inability or lack of prospects for controlling the future was also identified for instance; individuals identified uncertainty regarding their disease and symptom progression: “don’t know how fast my disease is going to progress. So the uncertainty of where I will be at what stage I will be in my disease… I don’t know, see, the uncertainty” [19] and individuals also identified an acknowledgement that nothing could be done. “Well, not really…If I thought there was anything I could be doing that would improve me I’d be doing it.” [21] | 5,8,10,11,16,17,19,21,23,28,29,31,32 |
|  | Negativity, pessimism, chronic sorrow and depression  *link to anxiety and negative emotions | Depression was consistently identified as a problem following the diagnosis of Parkinson’s disease. This may begin by the individuals not being able to feel positive about their diagnosis and it may be enhanced by feeling of being a burden on others, being overwhelmed by their situation and not being the same person they were before. One individual identified the impact of his depression “I quit work (because of Parkinson’s).I think I was probably depressed from then on until 2 or 3 months ago (about 4 years in duration).” [17]  Negative emotional responses to the illness were also apparent, including being upset, feeling numb or faint-hearted, for instance one participant stated “I have become very sensitive, I cry sometimes for small, silly things.” [12] | 3,11,12,17,19,21,27,28,30,32,37 |
|  | Difficulty or coming to terms with or Acknowledging the disease  *link with strategy of blocking | At diagnosis individuals could express difficulty in accepting the diagnosis, which could, also be represent by a complete denial. For instance, one individual stated “I was in denial for a couple of months after my diagnosis” [31], another stated I went into a real long denial process. Denial that I don’t want it. Put it that way. I knew I had it. But I didn’t want it. [17] and another individual stated “you want to live with it, but accepting it is difficult because you feel like you’re going mad” [36]  Individuals expressed difficulty in coming to terms with what had happened and the realisation that their diagnosis did not have a cure. Reactions initially included anger, shock, being upset, asking ‘why me?’. The resultant feelings following a diagnosis could be summarised by one women “I don’t know how other women have felt when they have been told but I found it very upsetting, felt very isolated with no backup.” [15]. Individuals could express a sense of mourning and a sense of future loss, of what they could have had. For some as more was learnt about the illness was a negative experience. | 9,15,16,17,20,21,24,25,31,36 |
|  | Acknowledgement and Acceptance | Adjustment was identified in different stages; including the recognition or acknowledgement that something is wrong, followed by the recognition that Parkinson’s disease could be the explanation. Diagnosis was identified by some individuals as a ‘relief’ as they could understand then what was happening to them. For instance one individual stated, “My whole family was sitting in her office[,] crying[,] saying something is wrong with me. She put us in touch with the head doctor at the clinic and he took a look at me and told me I had Parkinson’s. He sent me to a neurologist who confirmed it. It is the [same] story with everybody with PD and young [early] onset. You think you are going out of your mind. It was a huge relief to finally know what the problem was.” [20]. Following the diagnosis acceptance of having Parkinson’s was identified as an essential psychological stage in order to cope and live with the disease.  Being able to accept who the individual had become was also identified as important, one individual stated “It is important to accept yourself and the disease, you have to live with” [12]. Further to this it was identified that adjusting to an ever changing body was also important. Within these other needs of acceptance including an accepting the implications for one’s life, for instance, implications for one’s social roles, job role, or the stigma that can be generated from being in public. These broader considerations to adjustment may have represented more challenges for individuals. | 5,9,10,12,14,15,16,17,19,20,21,24,25,31,32,36 |

Note: 1 = Anderson, 2013, 2 = Andersson (2001), 3 = Banks(2006), 4 = Bingham (2006), 5 = Bramley (2014), 6 = Birgersson (2004), 7 = Buetow (2012), 8 = Capp-Ahlgren (2002), 9 = Charlton (2002), 10 = Davis (2004), 11 = Delaney (2012), 12 = Den Oudsten (2011), 13 = Drey (2012) , 14 = Elliott (2005), 15 = Flemming (2004), 16 = Haahr (2010), 17 = Habermann (1996), 18 = Habbermann (1999), 19 = Hermans (2011), 20 = Hodgson (2004) , 21 = Hurt (2012), 22 = Jones (2008), 23 = Liao (2013), 24 = Lindgren (1996), 25 = Marr (1991), 26 = Miller (2006), 27 = Mshana (2011), 28 = Oehlberg (2008), 29 = Olsson (2013), 30 = Soleimani (2014), 31 = Stanley-Hermanns (2010), 32 = todd (2010), 33 = Tolson (2002), 34 = Van Der Eijk (2011), 35 = Whitehead (2010), 36 = Benharoch (2004), 37 = Pretzer-Aboff (2009).

.

Table 3 Factors that influence the severity of the Interruption on individual’s life from Parkinson’s

| **Sub-theme** | **Code** | **Unit** | **Studies** |
| --- | --- | --- | --- |
| Illness related factors which effected individuals | Symptoms severity | Participants stated that the progression of the disease symptoms, as they became more severe, the ability to cope and manage independently was affected. The progression of the disease restricted the ability to multi-task e.g., walking and talking together.  Importantly, it was noted by individuals that recent deterioration could also cause similar problems. One further consequence of disease severity was the increase in mood disorders | 2,5,8,12,21,22,23,24,25,30 |
|  | fluctuation or Reversal of symptoms – link to medication  *link to autonomy  *link to stigma | Individuals identified “on” and “off” periods or fluctuations in functioning which severely influenced what individuals were able to achieve in daily life E.g., “I feel this disease is awful because when you have better days everyone thinks there is nothing wrong with you.” [3]. Some identified periods of time when they knew functioning would be worse or better whilst others suggested it wasn’t possible to identify this. E.g., “I don’t have any consistent days,” “My world revolves around a clock…the need to adjust daily activities according to their symptoms” [31]  This fluctuations causes problems with managing daily life for instance, “I have to wake him up and he has to help me to the  bathroom or I would just fall and hurt myself and fall off the toilet.” [15]. The fluctuations meant individuals had to plan and manage their day. | 2,3,8,9,12,13,15,16,21,22,29,31,36 |
| psychosocial and situational factors which challenge well-being | Difficulties of isolation | Individuals with spouses often had a source of support for basic activities they couldn’t do themselves, like shopping, which individuals without a spouse did not have.  Isolation was reported, and it an individual’s social life, roles and world generally retract in size and purpose. Some of the reasons for this included being tired and not feeling up to it, being cognitively impaired within interactions. Further to this individuals may also avoid social situations and could chose to isolate them self. For example, “I used to like to go out and sit somewhere taking alcohol with my friends, but now you cannot go out and sit with your friends because of the leg disability...and also you feel shy... you have no strength.” [27], worst case complete isolation was expressed for instance, “I’ve got no friends now. You lose all touch with people.” (I4) [35] | 2,10,12,23,25,27,28,29,30,35,36 |
|  | Limited autonomy, the burden on others and changing relationships and roles | There was general recognition that the ability to live independently was severely impaired, from basic task to more complex task, all aspects of living could be affects. One major problem could be the inability to drive which affected transportation.  The effects of an inability to engage in life are significant, for instance it prevents employment, social activities, and influences individuals roles and identities. Some individuals gave up trying for instance, ‘I realise the things I couldn’t do and didn’t waste energy trying to do them. [36]. Others utilised support, although this could be challenging, for instance a carer stated, “This morning, my wife had a shower. Taking a shower will cost her at least one hour; she became really slow. I find it very difficult to watch her; I notice she is having trouble with everything” [12]. Individuals with spouses often used them in a comprised way, like helping to a certain extent, for example even with intricate changes of sanitary pads. How acceptable assistance was varied from individual to individual. | 2,3,8,10,12,14,15,16,17,20,23,24,25,28,29,31,33,36 |
|  | Negative future thoughts and worries  *link to future identity and self  *link to confidence | The future could be a very daunting prospect for individuals with Parkinson’s; individuals were made very aware of prospects by seeing others with more advanced stages. For instance, examples of thinking about a negative and unwanted future included; “I have seen the future in the eyes, faces and activities, or in-activities, of my fellow Parkinson sufferers who are in more advanced stages of the disease than me.” [9] and these images led them to believe what they would become, a future where they wouldn’t want to live for instance, one individual=s stated he would be a ‘shivering wreck’ in 6 months’ time, while another stated ‘I can’t see a future’. [11]. This may represent aspects of chronic sorrow, since acceptance had taken place.  Much of responses about the future focused on fear about what they would become or no longer be able to do and also what they would lose in the future e.g., I have a sense of loss for my future with her (granddaughter) and any grandchildren I may or may not have.[15]. This feeling of loss could also cause individuals to think about how they would manage and cope in the future. E.g., *.” I know eventually I will end up in a wheelchair and I won’t be able to go anywhere, not without somebody pushing me…” [21].*  Individuals would identified fear of falling as a negative cognition, for instance one individual stated “I am afraid of falling. The shower is slippery. I also fell just the other day inside the bedroom. . . . My ribs hurt from the fall.” [37]. | 8,9,11,12,13,15,16,17,19,20,22,23,24,28,32,36 |
|  | Introducing negative behaviours  Impulse | Impulse control behaviours were reported in a number of studies. Individuals reported differently on how they made them feel for example some identified the process as being exciting, or gaining a feeling of euphoria or a ‘high’ and expressing that they didn’t want to control it. For instance, one individual stated the time when I started with the wrong thing [the ICBs] [ . . . ] and the kick I got out of doing it was also quite high. Strangely you get highs when you lose as well, if you lose a lot. (P9) [11].  However some stated the realised what they were doing was wrong but were not able to control it or stop doing it. For instance one individual stated “Well I did kind of know it were wrong, but I couldn’t stop it.” (P3) [11]. Gender differences were noted by this study, for example males tending to go in person to a gambling shop whilst females gambled on line. | 11 |
|  | Self-medicating | One study consider self-medication as a coping behaviour which enabled the participant to cope for instance : ‘Just casually popped the tablets whenever it suited my schedule’.[13] and ‘I wanted to time it so that I’d still got Sinemet in me when I went to bed to help me sleep all night. I had to argue for it. Now I’ve got my own little stash, low doses, single dose ones so I can take that if I wake. If I wake between two and four a.m. I’ll take an extra pill.” [13] | 13 |
|  | Difficulties of communication  -link to social confidence | Physical symptoms like rigidity or pain, physical changes in an individual voice such as the voice being husky, dull, mono-pitch, requiring throat clearing, combined with a slowness of speech, mumbling or having a tight mouth all influenced an individual’s ability to verbally communicate effectively. For instance, “I want to say something but something different comes out and I get awful confused” (F 67,  DD 10 years, HY 3, IM 50). [26]  Not being able to talk about what they want and this caused them to feel insecure, especially as they could know perfectly what they want to say e.g., “when I speak what comes out is fragmented and incoherent” [23]. This could also cause challenges within interactions for instance, I don’t like being in a shop asking for stuff because they’ll say ‘what do you want?’ and you have to keep  repeating yourself. It’s getting the right name of things out. (I4) [35] As a result individual could withdraw and stop wanting to communicate. | 1,3,12,20,23,26,29,31,35 |
| Factors affecting employment | Symptom severity | The symptoms of Parkinson’s made working very difficult for individuals, they were aware the adaptations were needed, that job security was threatened and individuals acknowledge that this would eventually lead to reduced hours working and then a job loss. | 3,12 |
|  | Age at diagnosis and effects on employment | Individuals who were younger would typically work longer than those who when diagnosed were older, although getting diagnosis nearer retirement meant individual may be able to ‘hang on’.  For some individuals their was resentment at having to work until they were considered by their employer too disabled, whilst other acknowledged that the symptoms of Parkinson’s made it challenging for employers to work with e.g., My timing is a bit erratic. I have so many clinics and appointments with Doctors and physios etc; I am hardly there. . .[3].  Finally, some individuals identify that knowing the law was important within a job after diagnosis. | 3,12 |
|  | Retirement and policy affecting individuals at their place of work and subsequent job availability | Various experiences relating to how individuals were treated because of their situation at work were reported, whilst some individuals identified that their situation was accounted for some suggested that it took time to implement, for instance, They] came up with this package of reducing my hours and they offered to provide me with a computer . . . I never actually took up any of that because I left before. . .[3]. Leaving work at an early age had financial implications although some reported receiving generous packages. One negative experience was reported “[I was] subjected to bullying treatment by first line Manager. Signed off with stress—quit after several weeks with no pension etc.” [3]  The possibility for retraining was limited by Parkinson’s, it was identified that computer skills could be one option however, this was not suitable for all. | 3 |
| Personal factors | Need for further resources | There was a call for greater long term care that is assisted with payment as well as need to recognise that individual were unable to pay for services them self. Individuals reported that the costs associated with Parkinson’s were high including the ability to afford treatment, which included transport, paying consultations and drugs | 12,27 |
| Environmental factors | Peaceful environments | Avoiding stress was identified as important and one participant stated “In the city there is a lot of noise. I like the silence.” [12] | 12 |
|  | Accessible and challenging environments | Individuals identified environmental factors which could challenge them, individuals identified that generally walking outdoors where there was the opportunity to for longer steps was more beneficial than having to take small steps indoors or around objects. For instance, one participant stated, ‘Inside it’s too crooked. You can’t get a straight run on it’ (20). Particular challenges included doorways, busy environments and environments that had poor disabled access. | 12,14,22 |
| Political | The rights and services available for individuals | Political changes were identified, including (1) the recent change for individuals with Parkinson’s to have the right to an ambulance. (2) uncertainty about the availability of health care services and a lack of resources which included, day centres, rehabilitation, home care, financial resources. One participant stated ”Its kind of pitiful that we don’t have outreach with physical therapy” [10] (3) a lack of outreach programmes or individuals that would contact them. | 12 |

Note: 1 = Anderson, 2013, 2 = Andersson (2001), 3 = Banks(2006), 4 = Bingham (2006), 5 = Bramley (2014), 6 = Birgersson (2004), 7 = Buetow (2012), 8 = Capp-Ahlgren (2002), 9 = Charlton (2002), 10 = Davis (2004), 11 = Delaney (2012), 12 = Den Oudsten (2011), 13 = Drey (2012) , 14 = Elliott (2005), 15 = Flemming (2004), 16 = Haahr (2010), 17 = Habermann (1996), 18 = Habbermann (1999), 19 = Hermans (2011), 20 = Hodgson (2004) , 21 = Hurt (2012), 22 = Jones (2008), 23 = Liao (2013), 24 = Lindgren (1996), 25 = Marr (1991), 26 = Miller (2006), 27 = Mshana (2011), 28 = Oehlberg (2008), 29 = Olsson (2013), 30 = Soleimani (2014), 31 = Stanley-Hermanns (2010), 32 = todd (2010), 33 = Tolson (2002), 34 = Van Der Eijk (2011), 35 = Whitehead (2010), 36 = Benharoch (2004), 37 = Pretzer-Aboff (2009).

.

Table 4 Cognitive, Physical and Spiritual Strategies and factors that influence an individual well-being

| **Theme** | **Code** | **Unit** | **Studies** |
| --- | --- | --- | --- |
| Cognitive and psychological approaches to aid functioning and well-being | Imagery of taste | One individual would imagine the taste of previously eaten foods | 2 |
|  | Giving the illness a name | One individual gave the disease a name “Parky” and into an entity and distinguishing it from the ‘self’ | 15 |
|  | Become ‘friends’ or interact with the illness | Studies identified the patients would become ‘friends’ with the illness and speak to it, e.g., tell it how to behaviour or hope you will cure.  E.g., “I became friends with the illness, and I speak with it, be good, I hope you will cure” [12] | 12,15 |
|  | Concentration, self-talk and the mental rehearsal of the movement | Previously autonomous skills required concentration for movement e.g., walking, waving at people, talking with people. Individuals had to think about the movement or interaction and consciously focus on making it occur. Individuals had to consider the position of their bodies and if all limbs were in the correct place when undertaking the movement.  E.g., “the mind had to control the body, instruct it, and try to get it to cooperate”. [17]  This could require more with dwindling effects of medication and could be associated with slowing down.  Mental rehearsal was required, which included thinking through the movement before it occurred, continuing the movement whilst it was being undertaken and correcting movement which was incorrect or out of synch e.g., size of steps and rhythm of movement in walking.  When the environment changed this meant being consciously aware of what to do in order to accommodate this e.g., coming up to steps when walking. E.g., “on approaching a doorway .. . I thought ‘‘smaller steps’’. You have got to say to yourself, ‘‘Stop, stop dead, take a long stride out’’’ (11). [22] | 10, 17,22,26,35 |
|  | Bringing psychological future or distance closer | Studies described the approach by some individuals of focusing on living now, living ‘one day at a time’, enjoying the moment or engaging in the present and not planning far in the future, or not worrying about the future. This was often driven by the uncertainty of the future due to the illness.  e.g., “well I try not worrying about the future. I try to live one day at a time. I just cope with it that way” [25] | 15, 16,17,19,20,25 |
|  | Making the best of each day  *one day at a time  *not dwelling on a problem | Individuals not hopeful for the future identified the need to make the best of their current situation, this meant doing what they were able to do given the disease limitation’s and managing as best they can, not dwelling on the problems, and dealing with change when it happened  e.g., “I did a ten minutes of ‘why me, why me?’ and then there’s no point in that and just got on with it. (I3) [35] and I mean you can lie in the bed and look at the walls and die, or you can manage as best you can. (Richard, P3) [32] | 4,5,11,16,17,19,20,25,31,32,35,36 |
|  | Expressing a positive outlook of having PD | Some individuals identify the importance of positivity, having a positive attitude and outlook and being positive towards the disease. Being positive was identified as an important coping strategy for maintaining as normal life as possible and overcoming a more depressive outlook.  e.g., “It’s like being in love, you have to learn to live with it and like what you feel like, like who you are, like everything about what you do and go on living.” [31] and ‘‘I sat down on a bench and thought: ‘this is shit’… then I agreed with myself that I had to be positive. I am not dead, I have only got Parkinson’s’’. [16]  and “some people wind up in suicide. But you have to change your way of thinking. Your have to think positively or if you think in a depressed manner, you’re beat” [25] | 5,9,10,12, 15,16,20,25,28,31,35 |
|  | Hope and optimism for the future | Maintaining hope being optimistic about the future and considering things in a positive and constructive way, seeing hope was seen as a central requirement of living and needed in order not to be beaten by the disease. There was hope that they could cope with what could happen.  E.g., I know that I won’t be cured. Intellectually I can understand that, but it feels like I will manage this. It’s going to be okay [29] | 4,12,16,20,29 |
|  |  | Hoping including hoping for a good outcome and having goals to achieve that, but also could including waiting or hoping for a cure. This hope was considered by some as needed to help them cope  E.g., Among all couples in this study, having hope was viewed as a positive coping strategy, particularly with regard to waiting for a cure. [20] and “There may be some miracle drug that will arrive…” [21]  And “having goals enabled them to go ahead and make the effort to carry on” [25] | 16,20,21,25, 28 |
|  | Appreciating being thankful for what the individual does have and the ability to cope | Individuals identified the importance of identifying positive aspects of one’s life; this could include aspects which were broader than the immediate effects of the disease. There was a focus on the positive aspects or assets in their life rather than deficits; often this was expressed as appreciating their current health and the ability to continue and handle activities. Thankfulness was mentioned on different levels e.g., being thankful for slow progression of the disease, being independent, having the correct diagnosis, not needed to work to support the family, having time with friends.  E.g., …I am blessed, I am blessed...that I am still able to attend church services and Sunday school…I thank that Lord every day that I still have the strength to take care of him.[4] and You don’t think you’ll end up like this and yet I’m grateful to be like this because I could be a lot worse. (NM1) [9] | 3,4,9,15,20,21,29 |
|  | Making comparison to others who have it worse | Individuals were able to make contrast to others in worst situations; the contrasts were made to those individuals who had significant physical disease, illness or impairment and often life limiting illnesses.  E.g., I have friends who have had lung problems, who have had heart attacks, total loss of vision, and are blind. We are alive” [10] or “We were glad it was not multiple sclerosis” [20]  Individuals also identified that their own illness could be worse, in that the symptoms of their illness could be worse or their general health could be worse e.g., “I feel that I’ve been blessed that I don’t have a lot of the…it seems like more traumatic symptoms” [14]. Additionally, individuals made comparisons to others who have Parkinson’s in a worse state.  Positive and negative comparisons could also be made the characteristic of others that made them stand out for instance, individuals who have the strength of character to cope were admirable in contrast to those who give up. | 5,9,10,14,15,20,25,31,35,36 |
|  | Humour | Individuals were able to have a sense of humour and ‘ make fun’ of themselves and their symptoms e.g., ‘‘here comes shuffle walk’’‘. [12] as well as situations that had occurred for instance, making humour about embarrassing social situations that had happened or experiences with strangers. | 5,12,20,26 |
|  | Retaining dignity | For some individuals it was important how other people saw them, this meant retaining a past identity and not letting the Parkinson symptoms intrude in a negative way on that identity. For instance, how they were seen by their grandchildren or being able to continue work around the symptoms of the illness.  It also meant being able to be dignified when out socially e.g., “I have to think to pull my trousers down, to pull my knickers down before I go to the toilet. Nine times out of ten I have already done the toilet.” [15] | 12,15,16,31 |
|  | Resilience and fighting spirit against previous adversity | Individuals could use distinct words that described positive strength of character, such as being a survivor, self-reliant, having exception determination which aided them positively in coping with the illness  E.g., “I’m stubborn in that respect and I don’t consider myself a patient yet.” [9]  Individuals spoke of not or resisting giving up, struggling, fighting or battling against the disease, exerting mental toughness against the disease, having perseverance and resolve to overcome the struggle, giving themselves pep talks to over-come the illness.  E.g., After bathing, I was unable to get out of the tub and was there for over two hours, no one  else was home. That’s life with Parkinson’s and that’s who I am. And I’ll take a bath next time with no reservations….You just gotta learn how to live with it. [31] | 5,9,12,14,15,24,25,26,29,31,32,37 |
|  | Not revealing diagnosis to others – *avoiding stigma  *hiding limbs  *isolating them self | Individuals could hide symptoms by concealing symptoms and not letting others seem them, for instance by stopping all movement, by hiding movement like having a leg under a desk. e.g., “I try and stop myself from moving. …so my fingers aren’t moving and my hands aren’t moving.” [5] or by using a car for transport. This could be assisted by using medication or timing outings when movement was more normal. It is possible this behaviour is affected by ones level of depression [21].  There were different reasons given for this e.g., some did not want others like their children to know realise or considering what the movement’s looked like e.g., on participant describe them as looking "abnormal" and resulted in feelings of shame or embarrassment [18]. Other reasons included being worried about losing one’s job or not wanting people to keep asking questions e.g., *'I try to go out less because that's all I hear*—*"what has happened to you?" [30]*. | 5,10,15,18,20,21,28,30,31 |
|  | Revealing the diagnosis to others | In contrast not revealing and hiding the symptoms, some individuals were honest and open to people, getting used to symptoms like thrashing because of the dyskinesias was reported. Some individuals identified that by allowing the symptoms in public allowed them to remain engaged with activities. | 18,35 |
|  | Using a distraction to stop thinking about the disease | Individuals would use occupations, leisure time activities or socialising as a way of distracting themselves from their symptoms and allowing individuals to feel good [14] about themselves or engage in meaningful activities. | 14,16,25 |
|  | Avoiding thinking about the disease  -link to normalcy | Individuals described a strategy of not thinking about the illness, or about their possible future, one reason for this is because it avoided the thoughts of the possible negative outcomes. E.g., ”change the subject in your brain” [24]. This process could be initiated during prognosis e.g., ‘you know it’s going to be terrible, you know, and er, I didn’t press it, ‘cos I didn’t really want to know’. (P7) [11]. A main reason for this strategy was to continue living life as normally as possible. | 5,9,11,18,24 |
|  | Seeking and learning more about the disease | A main form of being more autonomous was seeking information; this meant seeking resources like physical therapy [10] or the right doctor [20] as well as undertaking tasks to know more about the disease. At an extreme individual could keep diaries about their own condition and analyse what is happening. | 6,10,12,15,17,18,20 |
|  | Maintaining independence and autonomy | Individuals reported a need to be able to take care of them self and be independent and increase independence, not allowing others to take over, in order to retain control and empowerment in their own lives. E.g., “you don’t want someone to go ahead and do everything for you.. you need to do it for yourself” [25]. One participant stated that maintaining independence and activity was important to slow deterioration. [35]  Psychologically, individuals identified that they could identify what the disease means in their life, and not letting it have a dominating part of it. For instance one individual stated “I don’t ever call for help, really. I try to stay independent.”[37] | 12,15,16,18, 20,21,24,25,28,33,35,37 |
|  | Identifying what is manageable and planning ahead | Individuals identified a need to consider and plan activities or occupations that would be possible within the constraints of the illness. E.g., “I think ahead as to what it would be like and how I could handle it and I [may] go as far as how many people [will be there] and how long I will sit there” [14] and “We might do two or three things in the morning and then come home and have lunch and then we probably wouldn’t do anything in the afternoon.”[25]  Individuals had to consider what they could cope with, tolerate, what they energy levels would allow. Many individuals had to consider their “off” periods and adjust their day around medication and “on” time, when activity was possible. The disease introduced restriction, the need for rest or the need to slow down, as well as a certain level of uncertainty at not knowing its progression e.g., “So I live with Parkinson’s on a daily basis…I have to adjust my times…do it [daily activities] slowly or do it differently…I don’t know how fast my  disease is going to progress. [31]. This had implications for their occupation.  much of this mean spontaneity was restricted significantly and individuals were required to be organised, in order to know what needs to be done and complete tasks efficiently. This could extend to communication e.g., Yeah, I … tend to … work out my story before I start, I don’t do it ad lib, what I’ll say is this and before you came I thought I’ll say this … it never works out but I plan it.’ (I2) [35] | 5,10,12,14,16,17,21,25,29,31,35,36,37 |
|  | Changing value and importance of things | A few individuals highlighted that having Parkinson’s made them consider what was important in their day E.g., *‘….Now [I] really stop and think about what is important before [I] start the day.” [19]* | 19,28 |
|  | Looking to and attending the needs of others | Individuals considered the needs of others and the importance of keeping strong for others as a reason to keep going. However, for some, having Parkinson’s allowed them to look out to the needs of others. E.g., *It just made me a giver, I guess. It has made me a stronger person [19]*. Other ways of helping including working in an outreach service which helps individuals with Parkinson’s, starting a support group for Parkinson’s and volunteering. Individuals noted the benefits of this “*To start helping others who suffer has helped me to overcome reactive depression*”. [12] | 10,12,15,19,21,24,31 |
|  | Adapting life and engaging in Meaningful roles, activities and interactions–  -or to identity  -planning and purpose  *transcendence  -link with accomplishing tasks and sense of purpose | Engaging in meaningful interactions and activities, which were often central to an existing identity was important and provided a sense of purpose e.g., “I have some days that are more meaningful because I have events planned” [14]  Engagement in activities and interactions included leisure pursuits and hobbies, family, and enabling communities and groups to develop and support to be available. This didn’t just mean creating new opportunities for engagement it also meant fostering older engagement habits e.g., A chance to be able to carry on with former hobbies and interests is important. . .also it is important to be able to keep up with old friends. For instance, going for meals.’ [12]. Alternatively individuals could adapt how they worked in order to work for longer.  It is important to recognise that accomplishments and achievements created a sense of self-worth and had positive psychosocial consequences for participant’s well-being for instance increasing their self-efficacy and social confidence.  For some with Parkinson’s the diagnosis meant they now had opportunity to engage in activities that previously they were not able to. E.g., ‘I wanted to do it before, but I never had the time; one participant had started going to the gym another had begun to paint [36]. Further some individuals took the opportunity while they were able to undertake holidays or do things they had always wanted to. | 3,6,9,12,14,15,17,18,25,31,36 |
| Physical strategies that influenced well being | Retaining normalcy  *link to resilience and dignity  *link to control  *embracing the potential of social judgement or stigma  *link to identity | Individuals expressed an importance of living ‘normally’ or living as ‘usual’ meant engaging in activities that they used to and not letting the Parkinson’s disrupt their lives to a great extent. It was aided by the medication and by individual’s determination. The benefits were identified as being able to main confidence, social contact and a sense of purpose and self-worth. | 9,10,14,16,17,18,25 |
|  | Simplifying – cooking and eating | Simplifying cooking included using ready cooked food, having meals on wheels or having others support the cooking process.  In order to assist eating individuals would only use a spoon or a folk, use a larger glass or one with a handle, use their hand which could function better, lay the table in a more suitable way and remove items that could be looked e.g., “she lifted her glass with both hands to avoid spillage as her right hand trembled more than her left….if she chewed thoroughly and ate slowly, she managed.” [2] | 2,24 |
|  | Managing interactions | Individuals used certain strategies to aid communication such as keeping sentences short and manage, keeping silence more. Although this was only useful is the context and situation allowed for it. For instance, individuals identified that certain determinants influenced this like what he person was they were speaking to and their interest, the place time and context of the conversation. Others would just listen more e.g., “At one time I could keep a conversation going for ages but I can’t now, I just sit back and listen” (F 67, DD 10 years, HY 3,IM 50).[26]  Utilising other forms of communication like email, note writing or gestures. E.g., “Best thing invented was the email, if I make a mistake I can just cross it out” [26]  As a result withdrawing from communication to avoid the difficulties and using a spouse or close other to communicate in their place. | 26,28 |
|  | Managing actions and movement  *allowing more time  *changing or adapting activity | Managing movements meant keeping calm, waiting until the body was able to start again, telling themselves to maximise effort. The end result of this was that movement often took longer than before. …”I said if you’ll just let me take a 10-minute nap I’ll be ready to play cards. And so I did. I just got up, went and rested and they called me in 15 minutes and I was fine [14]. Understanding how their body responded to required time and learning.  Individuals were required to find alternative ways of undertaking movement of daily living e.g., *So I live with Parkinson’s on a daily basis…I have to adjust my times…do it [daily activities] slowly or do it differently* [19]. For instance, this could mean, not doing such fine motor skills, activities, finding a different way to exercise a movement e.g., getting the leg in the car with a swing, and leaving activities that could not be completed any more by using technology or someone else to do it instead.  Individuals mentioned techniques which aided walking and balance for instance undertaking shuffling movements to begin walking and prevent falling, or slowing down in order to continue walking e.g., “if you don’t stop, your shuffling will build up so much speed that you just fall. You can’t ignore it, you have to slow down” [10]. When fatigue became a problem some individuals would stop and rest, before starting walking again. | 9,10,12,14,18,19,21,26,28,29,31,36 |
|  | Using physical aids or assistive devices, adaptive aids and environmental adaptations  *link with changing ways of doing things | Physical aids and technology were used to enhance activity and reduce the need for particular movements. The aids included, having a walker or cane, *e.g., “walk with a walking stick sometimes just to get the rhythm to get your legs, long paces, rather than stuttering along” [21]* . Other aids included having an electric toothbrush, having home adaptations made like a walk in bath, wheelchair, weighted utensils, walkers with brakes, reach-grabber, safety belts, or Dosette boxes. Alternatively, for the purposes of a vocation individuals could use voice recognition software, a modified mouse or keyboard and a laptop. | 2,3,9,10,12,13,36,37 |
|  | Diet | Individuals identified that it could be important to vary when particular foods are consumed, as at particular times it may help the medication work more effectively e.g., “you need to have it [protein] more at night so that you’re ‘non-functioning to capacity time’ is just really when you’re sleeping”[14] more generally individuals identified the importance of a balanced diet and one individual considered the potential for alternative medicines to be of benefit e.g., “I went into the health food shop and they said to me about St. John’s Wort.” [15] | 14,15,17 |
|  | Using alternative therapies; exercise | A number of studies identified the potential benefits of exercise or yoga, far less studies considered psychological treatment such as seeing a psychologist. E.g., “I guess speaking to somebody, trying to resolve the problems. I currently am consulting a psychologist about my situation.” [28].  The physical activity was identified as something could help with their movement and better manage as a consequence, this was identified separately to any physical health benefits was suggested. Further to these benefits were identified with joining in with other people “Be more active, joining in activities.” [28] | 12,13,14,15,20,21,28,35,36 |
| Spiritual factors and strategies that influence well-being | Support from faith - accepting the illness, coping with illness and being hopeful | Individuals identified that their faith help individuals cope, deal with the consequences of the disease and have an ability to accept what had happened. A typical response was “The Lord will give me the health and strength I need to do the things that need to be done” [4]  Individuals suggested that faith helped them consider a sense of purpose in having the disease or the ability to view the situation as a blessing or look for positives within it, such that God was working for a greater good e.g., in helping others, for instance one patient stated “I really feel like I have this disease for a reason. I think there is a reason for it and I think I can help people who have it.” [20]  Importantly it provided a sense of have hope, a sense of control and a strength to carry on and preserve. For instance “We live by faith, not by sight” [19]. Further this, to focus on a relationship with God and to use this as a way of living *“So number one is to serve God. My relationship with God is really what gives me the daily strength.” [19] and “*I just say here I am, Lord, you know why.” [19]. Indeed, some stated that the process of surrendering to a higher power implied hope. | 4,6,19,20,24,30,31 |
|  | Interaction with God through prayer | Prayer was used for individual to seek guidance and assist them with how to manage their disease, what decisions to make (e.g., financial and personal) and to identified what activities to participate in. Some individuals also prayed for healing but also for feeling calmer or less stressed. | 4,9,12,13,27 |
|  | Support from faith and the impact on others | The above items relating to faith were strengthened and supported by faith communities which encouraged individuals. As one participant stated “We are able to encourage other people who are having problems such as this or similar problems…by our faith” [4] | 4,12 |

Note: 1 = Anderson, 2013, 2 = Andersson (2001), 3 = Banks(2006), 4 = Bingham (2006), 5 = Bramley (2014), 6 = Birgersson (2004), 7 = Buetow (2012), 8 = Capp-Ahlgren (2002), 9 = Charlton (2002), 10 = Davis (2004), 11 = Delaney (2012), 12 = Den Oudsten (2011), 13 = Drey (2012) , 14 = Elliott (2005), 15 = Flemming (2004), 16 = Haahr (2010), 17 = Habermann (1996), 18 = Habbermann (1999), 19 = Hermans (2011), 20 = Hodgson (2004) , 21 = Hurt (2012), 22 = Jones (2008), 23 = Liao (2013), 24 = Lindgren (1996), 25 = Marr (1991), 26 = Miller (2006), 27 = Mshana (2011), 28 = Oehlberg (2008), 29 = Olsson (2013), 30 = Soleimani (2014), 31 = Stanley-Hermanns (2010), 32 = todd (2010), 33 = Tolson (2002), 34 = Van Der Eijk (2011), 35 = Whitehead (2010), 36 = Benharoch (2004), 37 = Pretzer-Aboff (2009).

.

Table 5 Social support and Interactions that influenced well-being.

| **Sub-theme** | **Code** | **Sub-code** | **Unit** | **Studies** |
| --- | --- | --- | --- | --- |
| The use and benefits of social support | Provision of the different types of support or general acknowledgement of support | Family | Studies often mentioned social support in a general way; it was assumed that this included the different types of support. Families were frequently acknowledged as vital uses of social support. The support provided meant that individuals were united with others and could feel a sense of community and belonging, together with an enhanced quality of life. For instance, one individual stated, *“Support wise … I’ve too good a crew beside me, my wife is perfect, my daughter, my son, I couldn’t have anymore backing.” [21]* | 2,3,4,6,8,10,12,15,21,25,35,37 |
|  |  | Friends and others | Individuals valued close friends in a similar way to valuing family, as one individual stated they `brightened up the day' [2]. Thus there was a general acknowledgement that friends could provide satisfaction and aided their ability to cope. | 2,4,6,10,12,14,15,20,21,22,24,25,37 |
|  | Emotional support | Being valued by others | Individuals appreciated being accepting and not feeling judged or discriminated against by those who were around them. For instance, close family like children who valued them as a grandparent and not by the disease. Individuals needed to feel significant to others and valued by others. Being valued as unique was a critical component of care valued by participants. Identifying people who were understanding and considerate of the condition and making an effort to accommodate the individual was noted and highly valued for instance “wherever we go we get treated with special consideration. People really do make an effort”[9.]. individuals could be sensitive to other interest in them during conversations being wary of negative conversational cues for instance one stated on identifying the importance of eye contact “P:’It’s as if that they are seeing you as a whole person, not just writing down something about you while you are talking, they can see your body language when they look at you.” [12]. One individual also noted the importance of pets as a form of comfort and support. | 1,5,6,8,9,12,15,17,20,24,26,31,34 |
|  |  | Others being informed and knowing about Parkinson’s disease | Having others being informed about Parkinson’s was considered important in that it was perceived to reduce misunderstanding about individual’s behaviour’s, movements and interaction. Misunderstanding could occur with family as one individual stated *“I think sort of be more understanding, maybe my son, if I’m walking along the road he marches off and it’s only when I tell him to wait for me that, he doesn’t seem aware, he seems to think I can catch up and I get quite cross with that.” [21]*. Correcting interactions could be beneficial also on an individual’s stress and key in optimal in ensuring quality of life. One carer empathised patience as an essential quality, “Just patience, allow them to do what they can do. Let them show you some of what they can do; don’t take away their indepen­dence. When you take away their independence, even if it is no more than brushing their teeth or putting on a blouse, you are killing them. You are taking away their life. So be patient, and allow them to show you what they can do for themselves.” [37] | 12,17,21,22,24,37 |
|  | Belonging and unity with others who share a similar identity with their illness |  | Individuals identified a sense of belong and unity from being associated with other individuals who suffered with Parkinson’s. This was often generated within groups like support groups or rehabilitation sessions. Individuals could share similar feelings about the future and have solidarity with others identifying how they could progress forward. An important aspect of the group was it was a safe location to mix with others and socialise, for instance one individual stated “It would be good to have a place where to come together with others with the same condition, in order to share experiences and also to play, make jokes, etc [12]. Some could use such groups in a positive way to gain knowledge from others, to share experiences and be heard by someone, to consider how fortunate they are compared to others or how they could be inspired by others. An individual states the importance of the Parkinson’s group for him “I’m alright—say at Parkinson’s meetings—I can talk to them all day but … erm … I couldn’t sit and talk to somebody who I’d just met” [34].  For some, importantly comparisons with others could be negative and depression. | 6,9,10,12,13,15,16,20,21,25,28,29,31,34,35 |
|  | Tangible support |  | Tangible support was received in various forms, for example transportation could be supported by the local government, spouse or friends. Adaptions to the home could be received from the local government. Individuals like close family, or home help could support an individual by taking them out and doing tasks, such as shopping, cooking or other activities for them for example, one participant stated “My daughter took me round the [ice] rink and helped me round. She said ‘this is good physiotherapy’. And my son will come out and he’ll wash the car for me.” [15]. | 2,3,4,6,12,15,17,20,22,23,27,28,34 |
|  | Informational support |  | Informational support included access to information about the Parkinson’s disease, medication usage, as well as more general information needed for daily living. Informational support was gained through a number of sources included television programmes, the Parkinson’s charity, health care professionals, support groups, other people like family and even strangers as one individuals identified; ….”I had to figure out how I’m going to get there (to flight gate) because I can’t move the wheelchair with my right hand. So I looked up pleadingly to another fellow traveller and this women picked up my sign and she said “do you need help” and I said yes “yes ma’am”, I do” and she wheeled me down there and they couldn’t get me back to coach so I was treated to first class”….[17]. One individual noted the importance of the support “group I learn the most from the support group.” [20]. | 3,6,12,13,17,20,22 |
|  | Esteem support |  | Individuals highlighted the great value of esteem support from close others and family, it was identified as an importance source of motivation and encouragement and ability to continue in the face of difficulties. For instance one individual stated ”it is because of them that I have the courage to face this disease” [23] and another stated “Being with grandchildren feels good. Children are the best contacts, the children pep me up” [8] | 4,8,20,22,23 |
|  | Lack or inability of others to provide support - link with social perception |  | There was a number of reasons identified by participants why support could not be provided in an effective way, these included: (1) close others not being in a position to provide support for physical or emotional reasons or that changes in circumstances meant others were no longer available (2) feeling alienated and misunderstand by other people, not appreciated the functional limitations like ‘on’ or ‘off’ period whilst this could be for a variety of reasons for instance, one carer stated “we would go to church and he would say, I spoke to so and so and they walked past and this whole thing of being ignored, we realized later on is things that his voice had got so quiet’. [12], another example was suggested to result from assumptions made about an individual; “it is not a good feeling when people assume that you are too sick to participate” [12] (3) particular conflict was identified which caused distress for individuals and families, the could be not talking about their situation, arguing about it or worst case getting separated as a result of it. For instance, one individual noted “if I have trouble doing something and she comes along, she will finish it for me, which is all right sometimes, but she does it in such a way that [it]bothers me. Like “get out of the way and let me do it” she makes it sound like she could do it better and easier herself, so I don’t bother anymore” [17] . another individual noted “Well he’s a little bit deaf and he doesn’t really hear what I’m saying and he gets me so angry having to keep repeat myself, so I’m getting nastier, if you can’t hear me and I’ve got to keep repeating myself and I’m seizing up, what we are going to do? (I4) [35] | 3,6,8,12,14,15,17,20,21,24,26,28,30,32,35 |
| Experiences of the Interactions with the health service personnel | Staff understanding medication timing |  | Individuals reported both negative and positive experiences with health care professionals around understanding and using medication and timing of that medication. For instance one individual reported “Here’s your Miralax—for constipation.” And I said, “Excuse me, what are you giving him?” “Well, Miralax for constipation.” And I said, “Well no, that’s not—he doesn’t have constipation. He has Parkinson’s, and it better be Mirapex.” Another patients reported that a fall had occurred due to the wrong medication being dispensed at a local pharmacy. Individuals did however, report positive experiences, for instance with Parkinson’s disease nurses and their reliance on them for talking medication. Correct timing advice significantly improved individuals quality of life and well being as noted by one participant ‘I feel rosier taking it after breakfast . . . so I ‘phoned the nurse and she said (take it) about forty minutes before eating. It works better.’ (Respondent 12) [13] | 1,7,13 |
|  | Positive experiences with HCPs |  | Individuals who gave exceptional patient care and offered treatment and a sense of security from a large team network which could be accessed easily when needed. Individuals highlighted the benefit and value of a calm and confident approach “they [the staff on the Parkinson team] are positive, which makes me apprehend the situation positively— we are not in a hurry, and we do not work under time pressure, the doctor said, and I was so very happy” [6]. Individuals identified the importance of the team having respect for them and having a good relationship with the health care professional so sensitive or challenging issues could be discussed. One individual summarised their experience well stating “For me the neurologist provides the medical part of the treatment and the Parkinson nurse helps me to live with the disease. I can discuss everything with her. They have a separate, complementary role.” Female patient, 62 years old [34] | 1,6,12,13,15,20,21,30,34,37 |

Note: 1 = Anderson, 2013, 2 = Andersson (2001), 3 = Banks(2006), 4 = Bingham (2006), 5 = Bramley (2014), 6 = Birgersson (2004), 7 = Buetow (2012), 8 = Capp-Ahlgren (2002), 9 = Charlton (2002), 10 = Davis (2004), 11 = Delaney (2012), 12 = Den Oudsten (2011), 13 = Drey (2012) , 14 = Elliott (2005), 15 = Flemming (2004), 16 = Haahr (2010), 17 = Habermann (1996), 18 = Habbermann (1999), 19 = Hermans (2011), 20 = Hodgson (2004) , 21 = Hurt (2012), 22 = Jones (2008), 23 = Liao (2013), 24 = Lindgren (1996), 25 = Marr (1991), 26 = Miller (2006), 27 = Mshana (2011), 28 = Oehlberg (2008), 29 = Olsson (2013), 30 = Soleimani (2014), 31 = Stanley-Hermanns (2010), 32 = todd (2010), 33 = Tolson (2002), 34 = Van Der Eijk (2011), 35 = Whitehead (2010), 36 = Benharoch (2004), 37 = Pretzer-Aboff (2009).
